# Multicomponent integrated care for patients with chronic heart failure: systematic review and meta‐analysis

**DOI:** 10.1002/ehf2.14207

**Published:** 2022-11-14

**Authors:** Ya‐Feng Yang, Jia‐Xin Hoo, Jia‐Yin Tan, Lee‐Ling Lim

**Affiliations:** ^1^ Department of Medicine, Faculty of Medicine Universiti Malaya Kuala Lumpur Malaysia; ^2^ Department of Medicine and Therapeutics The Chinese University of Hong Kong Hong Kong SAR China; ^3^ Asia Diabetes Foundation Hong Kong SAR China

**Keywords:** Integrated care, Multifaceted approach, Chronic heart failure, Death, Hospitalization

## Abstract

To investigate the effectiveness of multicomponent integrated care on clinical outcomes among patients with chronic heart failure. We conducted a meta‐analysis of randomized clinical trials, published in English language from inception to 20 April 2022, with at least 3‐month implementation of multicomponent integrated care (defined as two or more quality improvement strategies from different domains, viz. the healthcare system, healthcare providers, and patients). The study outcomes were mortality (all‐cause or cardiovascular) and healthcare utilization (hospital readmission or emergency department visits). We pooled the risk ratio (RR) using Mantel–Haenszel test. A total of 105 trials (*n* = 37 607 patients with chronic heart failure; mean age 67.9 ± 7.3 years; median duration of intervention 12 months [interquartile range 6–12 months]) were analysed. Compared with usual care, multicomponent integrated care was associated with reduced risk for all‐cause mortality [RR 0.90, 95% confidence interval (CI) 0.86–0.95], cardiovascular mortality (RR 0.73, 95% CI 0.60–0.88), all‐cause hospital readmission (RR 0.95, 95% CI 0.91–1.00), heart failure‐related hospital readmission (RR 0.84, 95% CI 0.79–0.89), and all‐cause emergency department visits (RR 0.91, 95% CI 0.84–0.98). Heart failure‐related mortality (RR 0.94, 95% CI 0.74–1.18) and cardiovascular‐related hospital readmission (RR 0.90, 95% CI 0.79–1.03) were not significant. The top three quality improvement strategies for all‐cause mortality were promotion of self‐management (RR 0.86, 95% CI 0.79–0.93), facilitated patient–provider communication (RR 0.87, 95% CI 0.81–0.93), and e‐health (RR 0.88, 95% CI 0.81–0.96). Multicomponent integrated care reduced risks for mortality (all‐cause and cardiovascular related), hospital readmission (all‐cause and heart failure related), and all‐cause emergency department visits among patients with chronic heart failure.

## Introduction

Chronic heart failure (CHF) is a progressive and disabling disease with a high burden of morbidity and mortality. To date, 64.3 million people are living with heart failure globally.^1^ Heart failure is often associated with other co‐morbidities, making its management challenging as the majority of professional guidelines rarely address multi‐morbidity.^2^, ^3^, ^4^ This evolving epidemiology contributes to the increasing burden of CHF, especially in the aging population and regions of low‐to‐middle socio‐demographic index (SDI).^5^, ^6^, ^7^ In the USA, it was estimated the prevalence of CHF would increase by 46% from 2012 to 2030, affecting more than 8 million adults.^8^ In the past decades, despite a modest improvement in survival among patients with CHF, the mortality associated with CHF remains high.^9^

Inability to leverage existing healthcare resources, inconsistency in practice implementation, and insufficient integration of patient values and preferences are the key contributing factors to the slow progress in improving clinical outcomes among patients with CHF.^10^, ^11^, ^12^, ^13^ Despite the benefits of guideline‐directed medical therapy (GDMT) on clinical outcomes,^14^, ^15^, ^16^, ^17^ only up to 43% of patients with CHF received GDMT in real‐world practice.^16^, ^17^ These healthcare disparities not only impose negative impacts on clinical outcomes but also increase annual global healthcare expenditure at US$108 billion among patients with CHF.^18^

A quality teamwork benefits healthcare at different levels. These include organizational benefits (reduces unexpected admissions/costs of hospitalization), patients' benefits (improves quality of care and ensures patient safety), and healthcare team's benefits (enhances patient‐provider communication and integration of care).^19^, ^20^ Among patients with Type 2 diabetes, a meta‐analysis of 181 randomized clinical trials (RCTs) reported that multicomponent integrated care provided sustained benefits in the control of cardiometabolic risk factors.^21^ As CHF is usually associated with co‐morbidities such as Type 2 diabetes, obesity, and ischaemic heart disease,^22^ an effective multidisciplinary teamwork is of valuable importance. Herein, we conducted a systematic review and meta‐analysis to examine the effectiveness of multicomponent integrated care on mortality and healthcare utilization in patients with CHF.

## Methods

### Search strategy and study selection

The protocol was registered on PROSPERO (ID: CRD42020216275). We searched PubMed, EMBASE, Ovid, and the Cochrane Library for RCTs that were published from inception to 20 April 2022 using a combination of search terms (*Table* *S1*).

**Figure 1:**
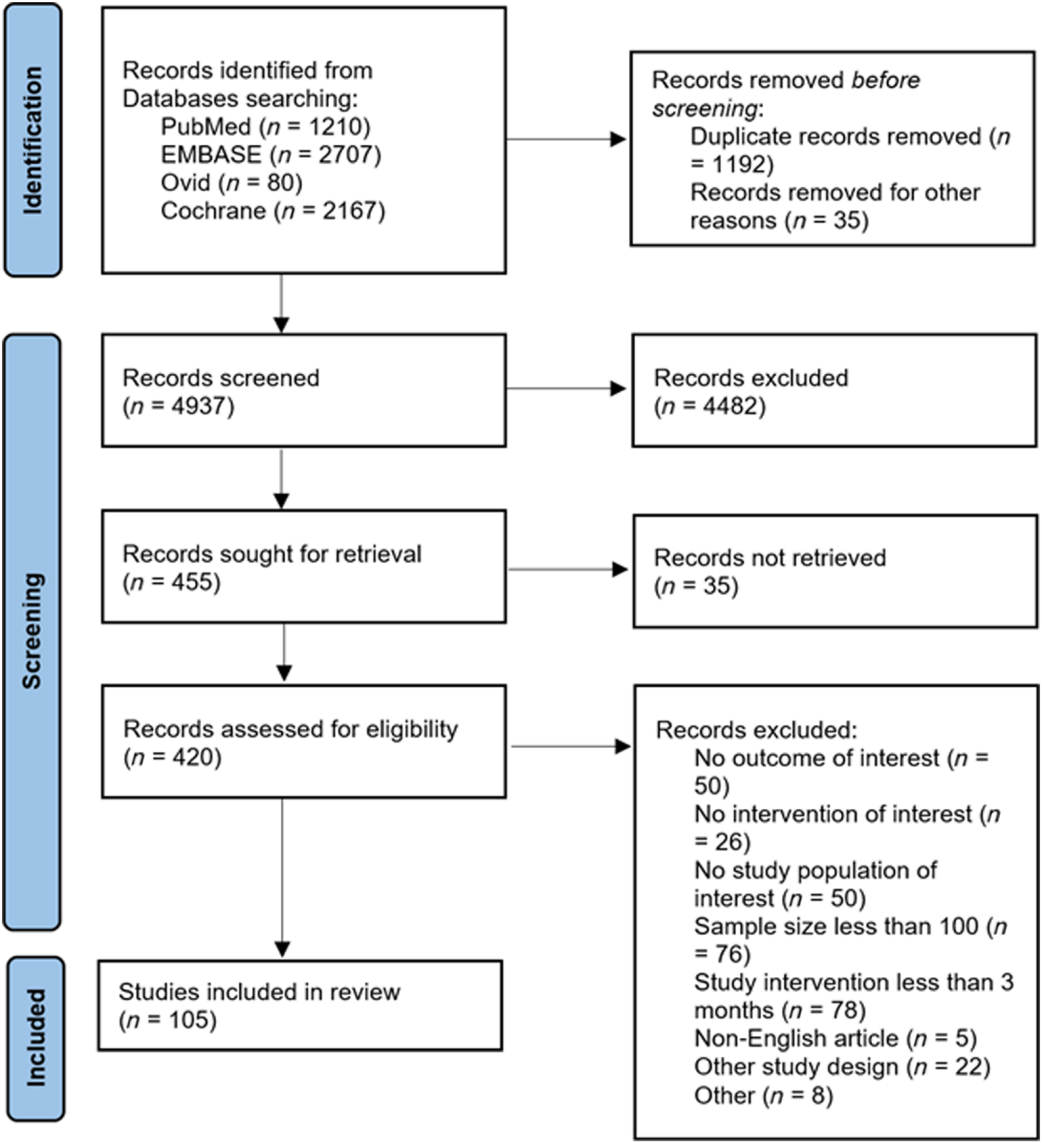
PRISMA flow diagram.

*Figure* *1* shows the PRISMA flow diagram. The eligibility criteria of RCTs were (i) patients aged ≥18 years, (ii) diagnosed with CHF either at the outpatient setting or discharged from inpatient stays, (iii) at least 100 patients, (iv) ≥12 weeks of intervention, and (v) published in English language. The primary outcome was mortality (all‐cause or cardiovascular), whereas the secondary outcomes included healthcare utilization (hospital readmission or emergency department visit).

We defined multicomponent integrated care as the implementation of two or more quality improvement (QI) strategies from different domains, namely, the healthcare system, healthcare providers, and patients.^21^ *Table* *S2* summarizes the definitions of all 13 QI strategies from three domains. We excluded RCTs that involved two or more QI strategies from a single domain.

### Data extraction and assessment of study quality

Two reviewers (YFY and JXH) independently screened the titles and abstracts. Relevant studies were reviewed in full text. Using a standardized case record form, we extracted data including the year of publication, country, study design, healthcare settings, sample size, duration and type of intervention, study population characteristics, and clinical outcomes. We used the Cochrane risk of bias tool to assess the quality of included RCTs for selection bias, performance bias, detection bias, attrition bias, reporting bias, contamination bias, and other biases.^23^ Either ‘low risk’, ‘high risk’, or ‘unclear risk’ was assigned for each criterion. Disagreement was resolved through discussion with a third reviewer (LLL).

### Statistical analysis

For binary outcomes, we used the Mantel–Haenszel method to calculate the risk ratio (RR) and 95% confidence interval (CI). For continuous outcomes, we calculated the standardized mean difference (SMD) with 95% CI using the inverse variance method. Trial data reported as median and interquartile range (IQR) were uniformly converted to mean ± standard deviation (SD) for continuous outcomes.^24^

Patients with CHF who received usual care as per the trial's routine practice were defined as the control group. There were seven trials that used an active comparator design.^25^, ^26^, ^27^, ^28^, ^29^, ^30^, ^31^ For analysis of individual QI strategy, we calculated the net differences of the number of individual QI strategies between the two groups. For six trials with a three‐arm design,^32^, ^33^, ^34^, ^35^, ^36^, ^37^ we examined the intervention differences between the study arm with the highest number of QI strategies and the comparison group. Financial incentive strategy was excluded as it was not reported in any of the included RCTs.

We used *I* ^2^ statistics to assess study heterogeneity. An *I* ^2^ of <40%, 40–60%, and >60% indicates no, moderate, and substantial heterogeneity, respectively.^23^ When either *P*‐value of the chi‐square test was <0.1 or the *I* ^2^ value was >50%, we used random‐effects models; otherwise, we selected fixed‐effects models to pool the RR. We also conducted a sensitivity analysis using random‐effects models for all outcomes. We assessed publication bias using the funnel plots and Egger's regression test.^38^ All analyses were conducted using RevMan version 5.4.1. A two‐tailed *P*‐value < 0.05 was considered statistically significant.

## Results

### Study characteristics

Of the 6164 RCTs screened, 105 RCTs^25^, ^26^, ^27^, ^29^, ^30^, ^31^, ^32^, ^33^, ^35^, ^36^, ^37^, ^39^, ^40^, ^41^, ^42^, ^43^, ^44^, ^45^, ^46^, ^47^, ^48^, ^49^, ^50^, ^51^, ^52^, ^53^, ^54^, ^55^, ^56^, ^57^, ^58^, ^59^, ^60^, ^61^, ^62^, ^63^, ^64^, ^65^, ^66^, ^67^, ^68^, ^69^, ^70^, ^71^, ^72^, ^73^, ^74^, ^75^, ^76^, ^77^, ^78^, ^79^, ^80^, ^81^, ^82^, ^83^, ^84^, ^85^, ^86^, ^87^, ^88^, ^89^, ^90^, ^91^, ^92^, ^93^, ^94^, ^95^, ^96^, ^97^, ^98^, ^99^, ^100^, ^101^, ^102^, ^103^, ^104^, ^105^, ^106^, ^107^, ^108^, ^109^, ^110^, ^111^, ^112^, ^113^, ^114^, ^115^, ^116^, ^117^, ^118^, ^119^, ^120^, ^121^, ^122^, ^123^, ^124^, ^125^, ^126^, ^127^, ^128^, ^129^, ^130^, ^131^, ^132^ (*n* = 37 607 patients with CHF) were eligible (*Figure* *1*). *Table* *S3* summarizes the study population characteristics, type of intervention, and study outcomes. The mean ± SD age was 67.9 ± 7.3 years, and 56.5% were men. The median duration of intervention was 12 months (IQR 6–12 months). The mean left ventricular ejection fraction (LVEF) was 37.7 ± 14.1%. The median number of QI strategies was 5 (IQR 4–6). The most common individual QI strategies implemented were case management (80%; *n* = 79 RCTs), continuous QI (70%; *n* = 65 RCTs), facilitated patient–provider communication (60%; *n* = 61 RCTs), and patient education (60%; *n* = 61).

Given the nature of the intervention wherein blinding of the study personnel and participants was not possible, 77% of the included RCTs had a high risk for performance bias (*Figure* *S1*). There was absence of publication bias for the majority of the clinical outcomes except for heart failure‐related and cardiovascular‐related mortality with slight asymmetry on funnel plots (*Figure* *S2*). Egger's test also did not suggest any evidence of publication bias for the outcomes tested (*Table* *S4*).

### Effects of multicomponent integrated care on mortality and hospital readmission

**Figure 2:**
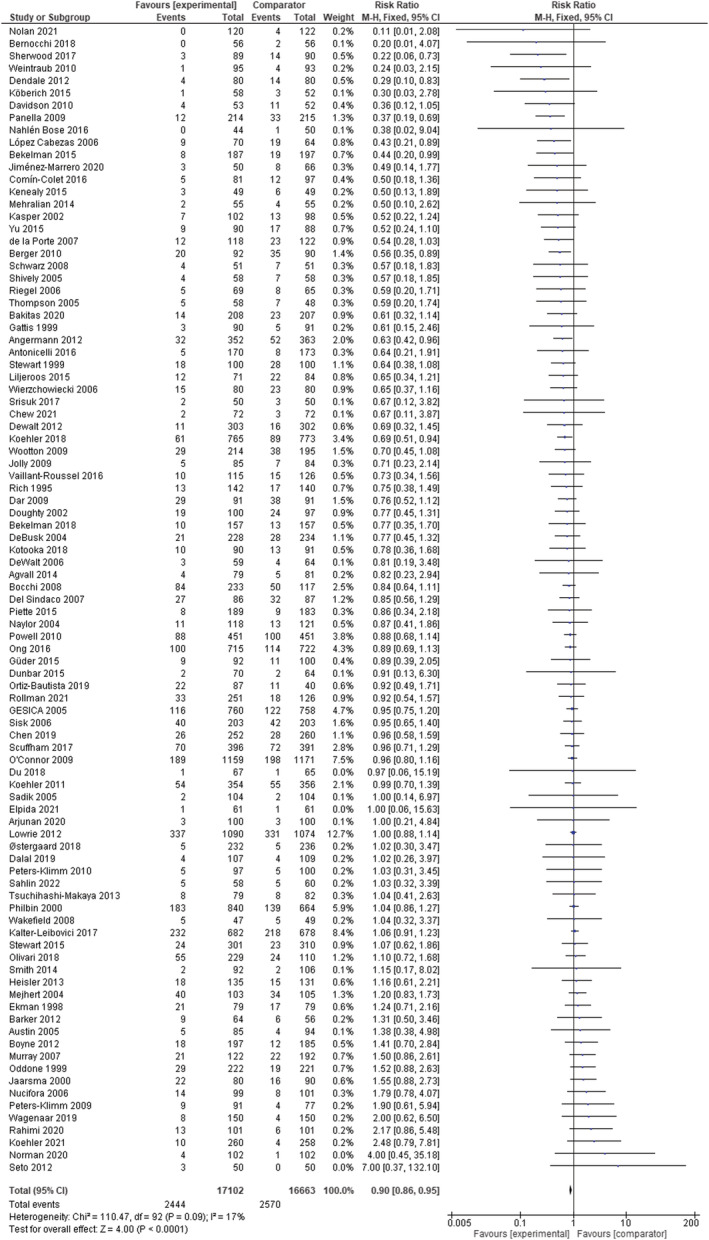
Meta‐analysis results of all‐cause mortality in patients with chronic heart failure (fixed‐effects model). CI, confidence interval; M‐H, Mantel–Haenszel.

Multicomponent integrated care was associated with reduced risk for all‐cause mortality (RR 0.90, 95% CI 0.86–0.95; *I* ^2^ = 17%; *n* = 93 RCTs) (*Figure* *2*). The corresponding RR for heart failure‐related mortality was 0.94 (95% CI 0.74–1.18; *I* ^2^ = 0%; *n* = 5 RCTs) (*Figure* *S3A*). Among 11 RCTs, the pooled RR for cardiovascular‐related mortality was 0.73 (95% CI 0.60–0.88; *I* ^2^ = 0%) (*Figure* *S3B*).

**Figure 3:**
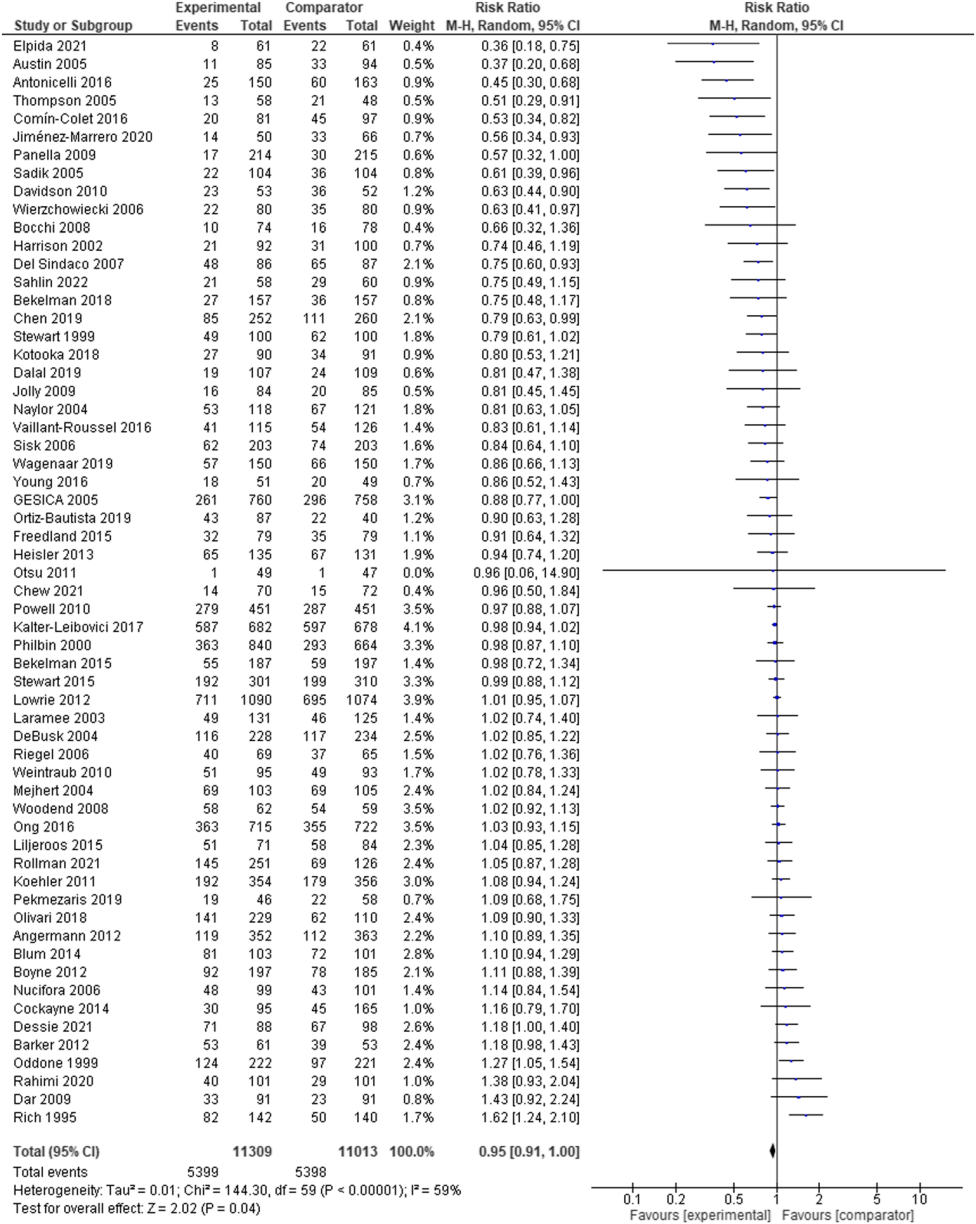
Meta‐analysis results of all‐cause hospital readmission in patients with chronic heart failure (random‐effects model). CI, confidence interval; M‐H, Mantel–Haenszel.

Regarding hospital readmission, the pooled RR was 0.95 (95% CI 0.91–1.00; *I* ^2^ = 59%; *n* = 60 RCTs) for any causes **(** *Figure* *3* **)**, 0.84 (95% CI 0.79–0.89; *I* ^2^ = 40%; *n* = 40 RCTs) for heart failure‐related cause (*Figure* *S3C*) and 0.90 (95% CI 0.79–1.03; *I* ^2^ = 54%; *n* = 17 RCTs) for cardiovascular‐related cause (*Figure* *S3D*). Compared with the usual care group, multicomponent integrated care was also associated with a reduced risk for all‐cause emergency department visits (RR 0.91, 95% CI 0.84–0.98; *I* ^2^ = 0%; *n* = 11 RCTs) (*Figure* *S3E*). In the sensitivity analysis, we reported consistent results for the aforementioned outcomes using the random‐effects models (*Figures* *S4* *and* *S5*).

*Figure* *S6A–D* shows the pooled standard mean difference (SMD) of the mean number of all‐cause, heart failure‐related, and cardiovascular‐related readmissions, as well as emergency department visits. When compared with usual care, multicomponent integrated care was associated with a lower number of heart failure‐related readmission (SMD −0.09; 95% CI −0.18 to −0.01; *P* = 0.03; *I* ^2^ = 41%) and emergency department visit (SMD −0.12; 95% CI −0.23 to −0.02; *P* = 0.02; *I* ^2^ = 0%). There was no statistically significant difference in the number of all‐cause readmission (SMD −0.04; 95% CI −0.10 to 0.02; *P* = 0.17; *I* ^2^ = 37%) and cardiovascular‐related readmission (SMD −0.06; 95% CI −0.14 to 0.01; *P* = 0.09; *I* ^2^ = 19%). The sensitivity analysis using random‐effects models also showed consistent results (*Figure* *S7A–D*).

### Effects of individual QI strategy on mortality and hospital readmission

**Figure 4:**
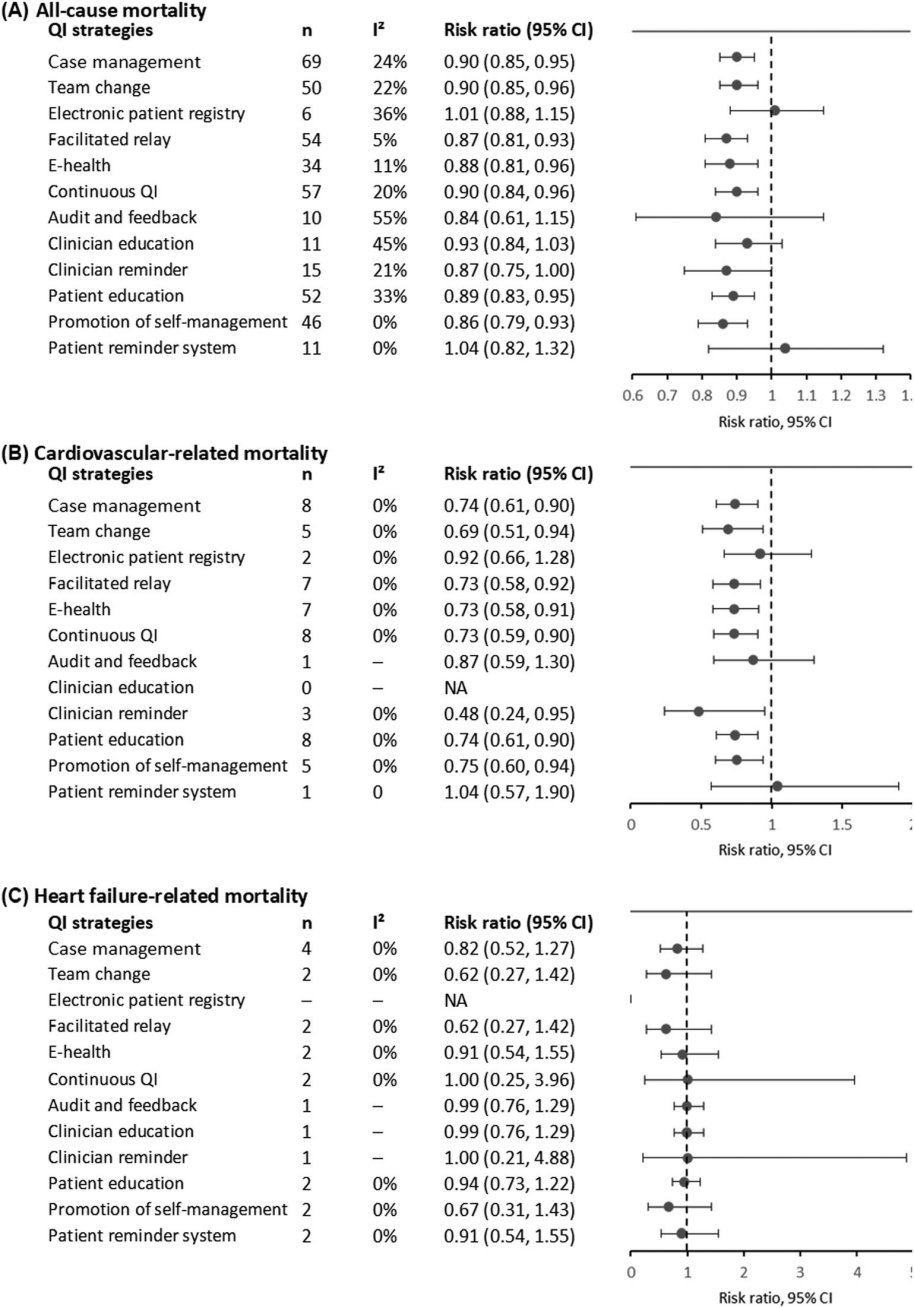
Effects of multicomponent integrated care on all‐cause mortality (A), cardiovascular mortality (B), and heart failure‐related mortality (C) in patients with chronic heart failure. CI, confidence interval; e‐health, electronic health; QI, quality improvement (fixed‐effects model was used when *I* ^2^ < 50%; otherwise, random‐effects model was used).

The top three QI strategies in reducing risk for all‐cause mortality were promotion of self‐management (RR 0.86, 95% CI 0.79–0.93, *I* ^2^ = 0%; *n* = 46 RCTs), facilitated patient–provider communication (RR 0.87, 95% CI 0.81–0.93, *I* ^2^ = 5%; *n* = 54 RCTs), and e‐health (RR 0.88, 95% CI 0.81–0.96, *I* ^2^ = 11%; *n* = 34 RCTs) (*Figure* *4* *A* and *Table* *S5*).

For cardiovascular‐related mortality, clinician reminder (RR 0.48; 95% CI 0.24–0.95; *I* ^2^ = 0%; *n* = 3 RCTs), team change (RR 0.69, 95% CI 0.51–0.94; *I* ^2^ = 0%; *n* = 5 RCTs), facilitated patient–provider communication (RR 0.73, 95% CI 0.58–0.92; *I* ^2^ = 0%; *n* = 7 RCTs), e‐health (RR 0.73, 95% CI 0.58–0.91; *I* ^2^ = 0%; *n* = 7 RCTs) and continuous QI (RR 0.73, 95% CI 0.59–0.90; *I* ^2^ = 0%; *n* = 8 RCTs) were the most effective QI strategies (*Figure* *4* *B* and *Table* *S5*). For heart failure‐related mortality, there were only up to four RCTs that we could analyse for individual QI strategies but the results were not significant (*Figure* *4* *C* and *Table* *S5*).

**Figure 5:**
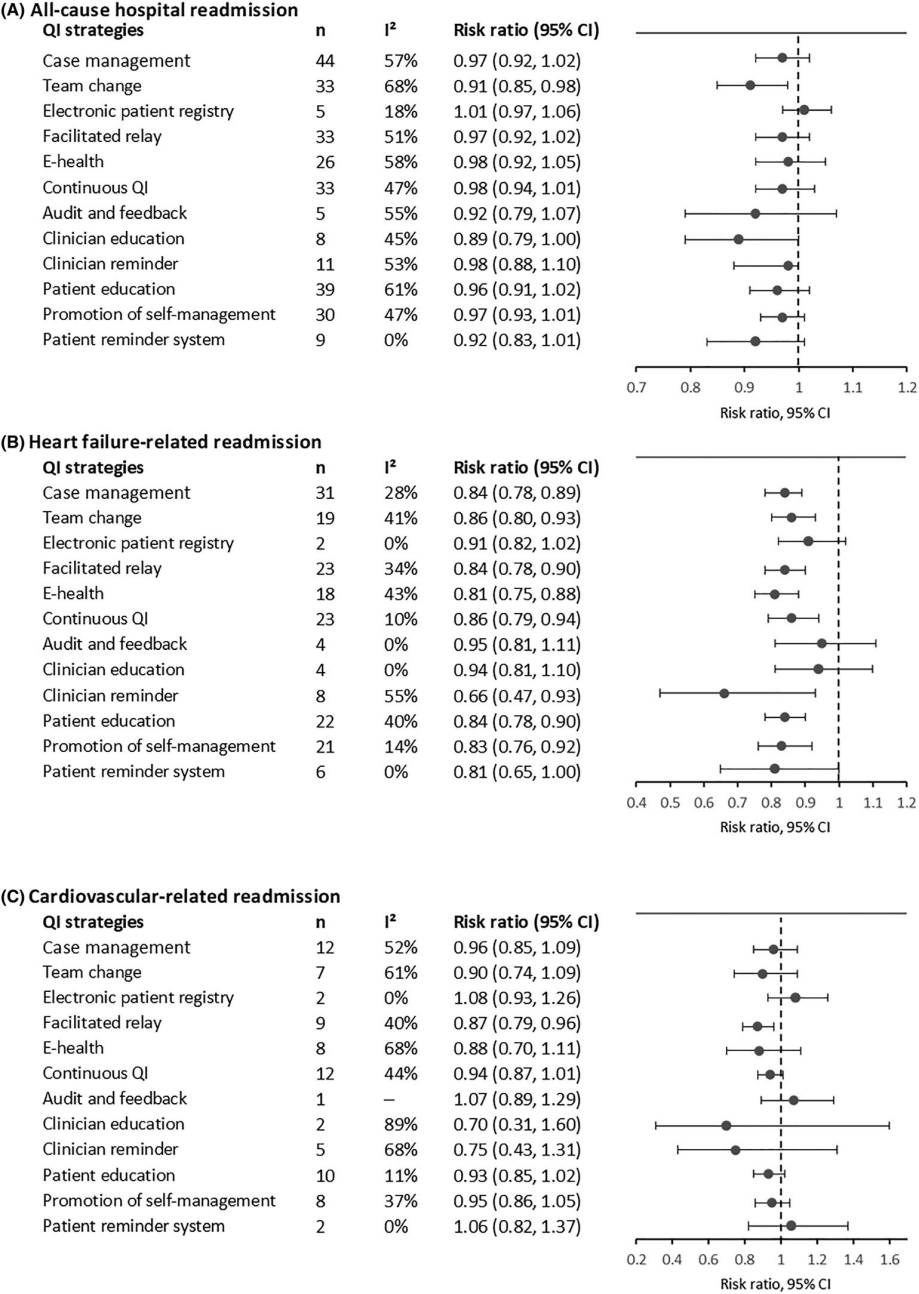
Effects of multicomponent integrated care on all‐cause hospital readmission (A), heart failure‐related readmission (B), and cardiovascular‐related readmission (C) in patients with chronic heart failure. CI, confidence interval; e‐health, electronic health; QI, quality improvement (fixed‐effects model was used when *I* ^2^ < 50%; otherwise, random‐effects model was used).

For all‐cause hospital readmission, team change (RR 0.91, 95% CI 0.85–0.98; *I* ^2^ = 68%; *n* = 33 RCTs) and clinician education (RR 0.89; 95% CI 0.79–1.00; *I* ^2^ = 45%; *n* = 8 RCTs) were the most effective QI strategies (*Figure* *5* *A* and *Table* *S5*). Eight out of 12 QI strategies showed their effectiveness in improving heart failure‐related readmission (*P* < 0.05) except for electronic patient registry, audit and feedback, clinician education, and patient reminder system (*Figure* *5* *B* and *Table* *S5*). For cardiovascular‐related readmission, facilitated patient–provider communication (RR 0.87; 95% CI 0.79–0.96; *I* ^2^ = 40%; *n* = 9 RCTs) showed significant association (*Figure* *5* *C* and *Table* *S5*).

Facilitated patient‐provider communication (RR 0.88; 95% CI 0.80–0.97; *I* ^2^ = 0%; *n* = 8 RCTs) and continuous QI (RR 0.89; 95% CI 0.81–0.97; *I* ^2^ = 8%; *n* = 7 RCTs) were associated with a reduced risk for emergency department visit (*Figure* *S8A*).

### Effects of multicomponent integrated care on medication use

We reported the subgroup analysis (baseline vs. post‐intervention) on the changes of use of renin–angiotensin system (RAS) blockers, β‐blockers, aldosterone antagonist, and diuretics (*Figure* *S9*). The use of RAS blockers showed no significant changes between the intervention and usual care groups (*P* = 0.080). On the other hand, compared with the usual care group, the intervention group reported an increased use of β‐blockers (RR 1.03, 95% CI 1.01–1.05; *I* ^2^ = 41%, *n* = 23 RCTs), aldosterone antagonist (RR 1.09; 95% CI 1.03–1.15; *I* ^2^ = 3%; *n* = 15 RCTs), and diuretics (RR 1.02, 95% CI 1.00–1.04; *I* ^2^ = 22%, *n* = 12 RCTs).

## Discussion

In the present meta‐analysis of 105 RCTs, implementation of multicomponent integrated care for 3 months or more was associated with a 10–30% reduced risk for mortality (all‐cause and cardiovascular related), hospital readmission (all‐cause and heart failure related), and all‐cause emergency department visits among patients with CHF. These benefits were observed in addition to usual care. Previously published meta‐analysis (i) included fewer studies and/or a mixture of RCTs and observational cohort studies, (ii) reported fewer subtypes of clinical outcomes (mainly all‐cause mortality and hospital readmissions), and (iii) focused on single health domain while the present meta‐analysis used a stricter definition of multicomponent integrated care.^133^, ^134^, ^135^, ^136^, ^137^, ^138^ To our knowledge, the present meta‐analysis has provided the most comprehensive assessment of the efficacy of different QI strategies in preventing both mortality and healthcare utilization among patients with CHF.

Among different QI strategies, patient self‐management, facilitated patient–provider communication, team change, clinician reminder, and e‐health were the most effective QI strategies to reduce risk for mortality among patients with CHF. These findings were broadly aligned with a meta‐analysis of multicomponent integrated care on cardiometabolic risk factors among patients with Type 2 diabetes.^22^ Of note, clinician reminder and e‐health were found to be effective among patients with CHF in the present meta‐analysis, but not reported in those with Type 2 diabetes, which warrants attention.^21^

Clinician reminder system can be an effective tool to provide support and assistance to the healthcare providers for improving care delivery. Its effectiveness among patients with CHF depends on the clinical context.^139^ In the present meta‐analysis, the most common approaches in delivering clinician reminder were sending alert message via email, phone message, alerts via electronic medical system, or an online platform. In primary care setting, clinician reminder system plays an important role in providing evidence‐based information and latest reminder to the healthcare providers for further action. A review involving 30 RCTs and 5 non‐RCTs reported a 6.5% improvement (IQR 3.8–17.5%) on their compliance with preventive guidelines and compliance with disease management guidelines when using computer‐generated clinician reminders delivered on paper, although the effect on clinical outcomes was unclear.^140^ Another review reported modest effects of computerized prompts on clinicians' prescribing behaviour wherein overall prescribing improved from 28% to 31% in 13 different areas.^141^ In line with our results, a clinician reminder and alert system can be an effective approach in the management of CHF to provide support and assistance to the healthcare providers in improving care.

In the present meta‐analysis, the commonly implemented e‐health tools were telemonitoring (e.g. remote monitoring through phone calls, electronic medical devices, online platforms, and mobile applications) and e‐learning (e.g. videotapes, short message service, and video/audio conferences). Other studies involving patients with chronic kidney disease revealed the benefits of e‐health in promoting patient empowerment and self‐care efficacy.^142^, ^143^, ^144^, ^145^, ^146^ Of note, this approach is not constrained by face‐to‐face delivery of information, education, or clinical monitoring, an attractive feature that is very useful in the COVID‐19 pandemic.

RAS blockers/angiotensin receptor neprilysin inhibitors, β‐blockers, aldosterone antagonists, and sodium‐glucose co‐transporter‐2 inhibitors are the four pillars of CHF management, which can prevent mortality and reduce healthcare utilization.^147^ In the present meta‐analysis, we reported a significant increase in the use of β‐blockers, aldosterone antagonists, and diuretics among patients with CHF with implementation of multicomponent integrated care. By contrast, the proportion of patients receiving this GDMT reduced in the usual care group. The proportion of patients treated with RAS blockers was already high at baseline; therefore, we were unable to observe a significant improvement after the implementation of multicomponent integrated care. A review that involved 25 low‐income and middle‐income countries (LMICs) reported the suboptimal use of GDMT in patients with CHF, with only 34% and 32% of patients treated with β‐blockers and aldosterone antagonists, respectively.^148^ Therefore, the present meta‐analysis showed that multicomponent integrated care can close the care gaps and optimize the use of GDMT in patients with CHF in real‐world settings.

We acknowledged several study limitations. First, there were RCTs in which details of the intervention and usual care groups were not clearly described. This created challenges to assess the complexity and intensity of study interventions. Second, due to limited data, we were unable to conduct subgroup analyses by the type of heart failure, namely, heart failure with preserved ejection fraction (HFpEF), mildly reduced ejection fraction (HFmrEF), and reduced ejection fraction (HFrEF), as well as by the status of co‐existing chronic kidney disease. Third, a low number of RCTs in the present meta‐analysis that captured heart failure‐related mortality as a key clinical outcome could potentially explain its insignificant results. Of note, heart failure becomes the centre‐stage in chronic disease management after the positive results of the EMPA‐REG Outcome trial in 2015.^149^ In the present meta‐analysis, only two out of five RCTs analysed were published after 2015 with a low number of heart failure events. Fourth, given a lack of individual‐level data, we were not able to examine the factors contributing to cardiovascular‐related hospital readmissions such as coronary artery disease, valvular dysfunctions, and hypertensive emergency. Besides, future RCTs should consider using the length of stay as a surrogate clinical marker for assessing the efficacy of multicomponent integrated care in CHF. Last, heterogeneity related to the study population characteristics and outcome measures might lead to reporting bias. To address this, we have conducted random effects models, and our results confirmed the clinical benefits of multicomponent integrated care.

## Conclusions

Multicomponent integrated care can reduce mortality (all‐cause and cardiovascular related), hospital readmission (all‐cause and heart failure related), and all‐cause emergency department visits among patients with CHF. Future research on patient‐reported outcomes and cost‐effectiveness of multicomponent integrated care is needed before its large‐scale implementation especially in LMICs.

## Conflict of interest

No potential conflict of interest relevant to this article was reported.

## Funding

This work was supported by the UK–Malaysia Joint Partnership on Non‐Communicable Diseases under the Malaysia Partnership and Alliances in Research (MyPAiR), funded by the Ministry of Education, Malaysia, and Medical Research Council, United Kingdom (Grant Number: IF076‐2019). The funders did not have any role in the design, interpretation of the study, or the decision to publish the results.

## Supporting information


**Figure S1.** Risk of bias assessment.
**Figure S2.** Funnel plots of publication bias in the meta‐analysis.
**Figure S3.** Forest plots of heart failure‐ and cardiovascular‐related mortality and hospital readmissions in patients with chronic heart failure.
**Figure S4.** Meta‐analysis results of all‐cause mortality in patients with chronic heart failure using the random‐effects models.
**Figure S5.** Forest plots of heart failure‐ and cardiovascular‐related mortality and hospital readmissions in patients with chronic heart failure using the random‐effects models.
**Figure S6.** Forest plots of the number of events (mortality and hospital readmissions) using the fixed‐effects models.
**Figure S7.** Forest plots of the number of events (mortality and hospital readmissions) using the random‐effects models.
**Figure S8.** Effects of individual quality improvement strategies on healthcare utilization in patients with chronic heart failure.
**Figure S9.** Use of medication therapy from baseline to the end of intervention in patients with chronic heart failure.
**Table S1.** Search strategy for meta‐analysis and systematic review.
**Table S2.** Definitions of quality improvement strategies in chronic heart failure from previous meta‐analysis (1).
**Table S3.** Key characteristics of the 105 included randomised clinical trials.
**Table S4.** Assessment of publication bias using the Egger's test.
**Table S5.** Meta‐analysis results of mortality and hospital readmission, stratified by individual quality improvement strategy.
