# Supplementary material for: Multicomponent integrated care for patients with chronic heart failure: systematic review and meta‐analysis
Source: ESC Heart Fail. 2022 Nov 14;10(2):791–807. doi: 10.1002/ehf2.14207 (PMC10053198; doi:10.1002/ehf2.14207)
Supplement: Supplementary file 1 — Figure S1. Risk of bias assessment. Figure S2. Funnel plots of publication bias in the meta‐analysis. Figure S3. Forest plots of heart failure‐ and cardiovascular‐related mortality and hospital readmissions in patients with chronic heart failure. Figure S4. Meta‐analysis results of all‐cause mortality in patients with chronic heart failure using the random‐effects models. Figure S5. Forest plots of heart failure‐ and cardiovascular‐related mortality and hospital readmissions in patients with chronic heart failure using the random‐effects models. Figure S6. Forest plots of the number of events (mortality and hospital readmissions) using the fixed‐effects models. Figure S7. Forest plots of the number of events (mortality and hospital readmissions) using the random‐effects models. Figure S8. Effects of individual quality improvement strategies on healthcare utilization in patients with chronic heart failure. Figure S9. Use of medication therapy from baseline to the end of intervention in patients with chronic heart failure. Table S1. Search strategy for meta‐analysis and systematic review. Table S2. Definitions of quality improvement strategies in chronic heart failure from previous meta‐analysis (1). Table S3. Key characteristics of the 105 included randomised clinical trials. Table S4. Assessment of publication bias using the Egger's test. Table S5. Meta‐analysis results of mortality and hospital readmission, stratified by individual quality improvement strategy. [file EHF2-10-791-s001.docx]

**Multicomponent integrated care for patients with chronic heart failure: systematic review and meta-analysis**

**Supporting information**

**Figure S1.** Risk of bias assessment


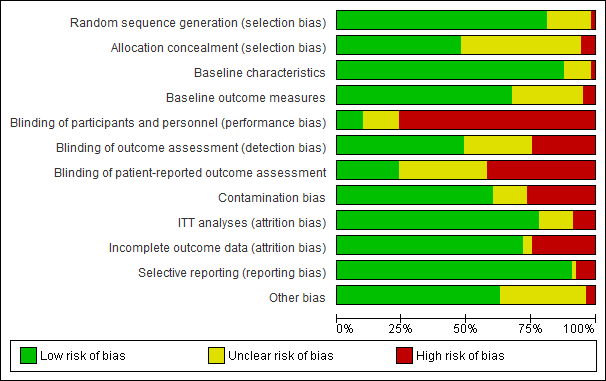


**Figure S2.** Funnel plots of publication bias in the meta-analysis


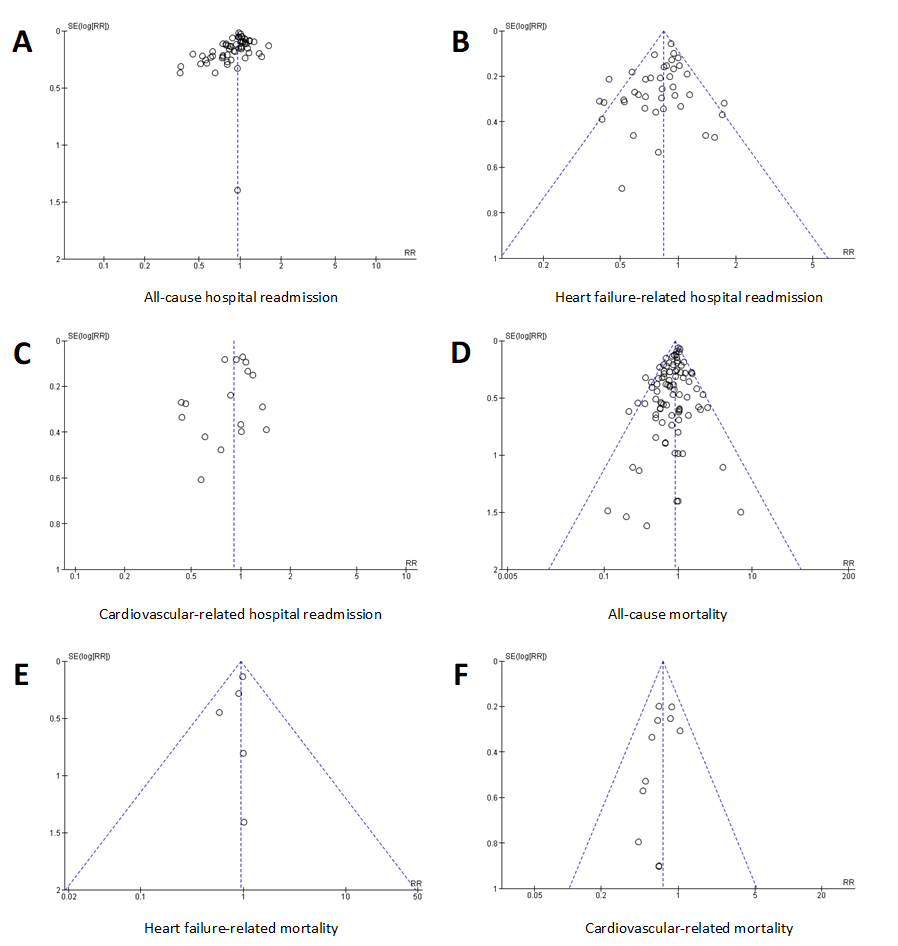


Footnotes: SE, standard error; MD, mean difference; RR, relative risk; HF, heart failure; vs, versus.

**Figure S3.** Forest plots of heart failure- and cardiovascular-related mortality and hospital readmissions in patients with chronic heart failure

Footnotes: CI, confidence interval; M-H, Mantel-Haenszel. (Fixed-effects model was used when *I^2^*<50%, otherwise, random-effects model was used)

**Figure S4.** Meta-analysis results of all-cause mortality in patients with chronic heart failure using the random-effects models


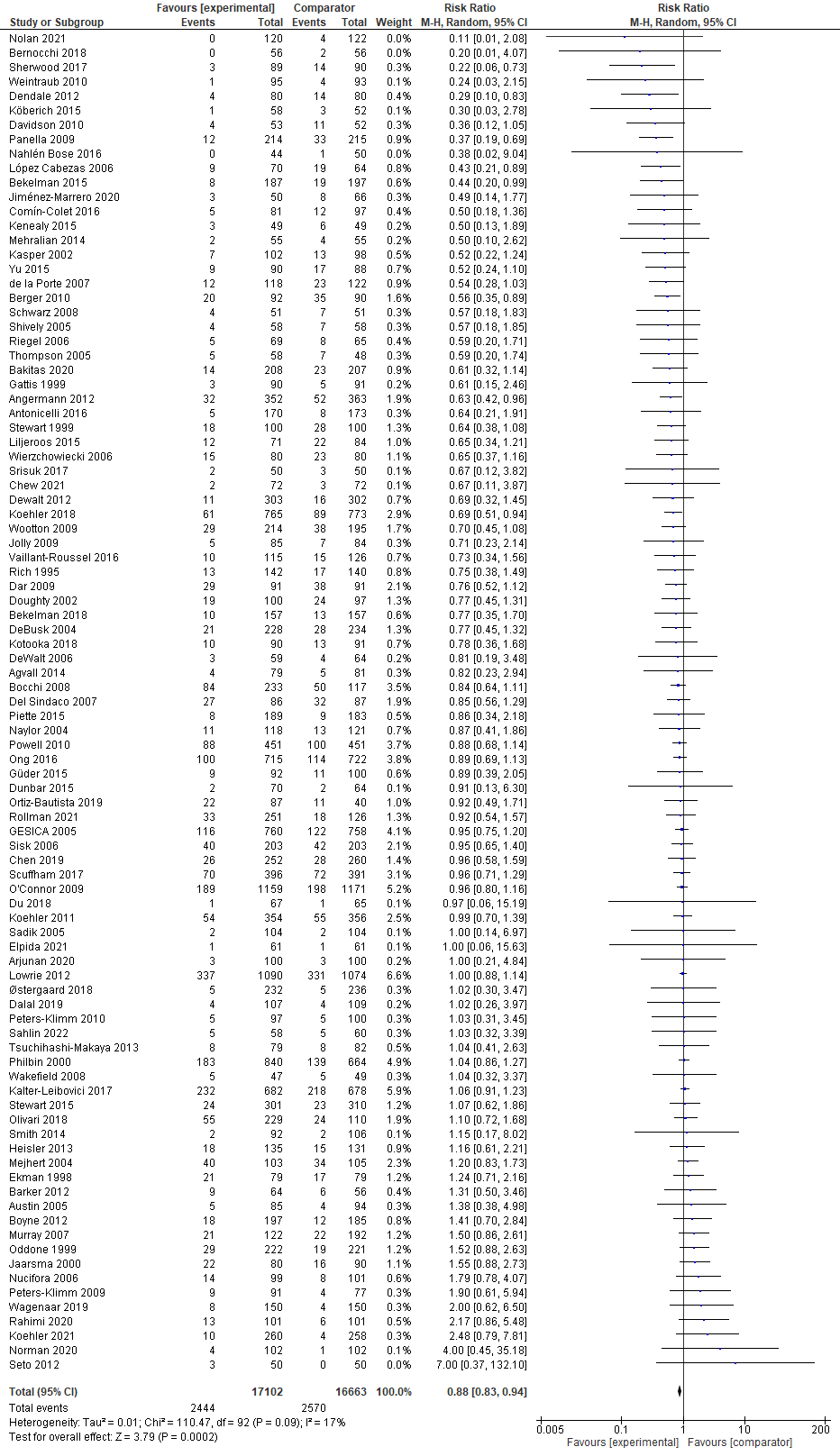


Footnotes: CI, confidence interval; M-H, Mantel-Haenszel

**Figure S5.** Forest plots of heart failure- and cardiovascular-related mortality and hospital readmissions in patients with chronic heart failure using the random-effects models

Footnotes: CI, confidence interval; M-H, Mantel-Haenszel

**Figure S6.** Forest plots of the number of events (mortality and hospital readmissions) using the fixed-effects models


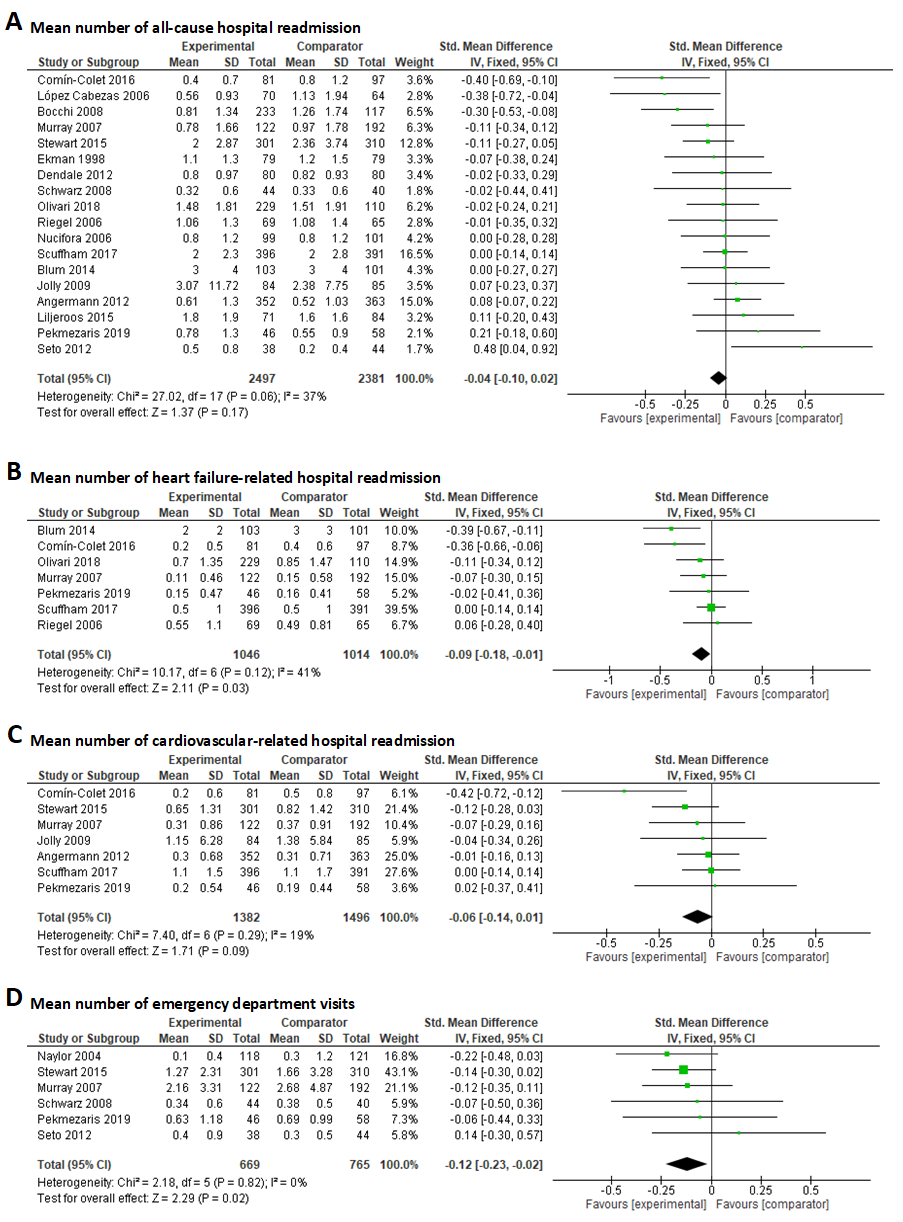


Footnotes: Std, standard; IV, inverse variance; CI, confidence interval; SD, standard deviation

**Figure S7.** Forest plots of the number of events (mortality and hospital readmissions) using the random-effects models

Footnotes: Std, standard; IV, inverse variance; CI, confidence interval; SD, standard deviation

**Figure S8.** Effects of individual quality improvement strategies on healthcare utilization in patients with chronic heart failure

Footnotes: CI, confidence interval; E-health, electronic health; QI, quality improvement; ED, emergency department. (Fixed-effects model was used when *I^2^*<50%, otherwise, random-effects model was used)

**Figure S9.** Use of medication therapy from baseline to the end of intervention in patients with chronic heart failure


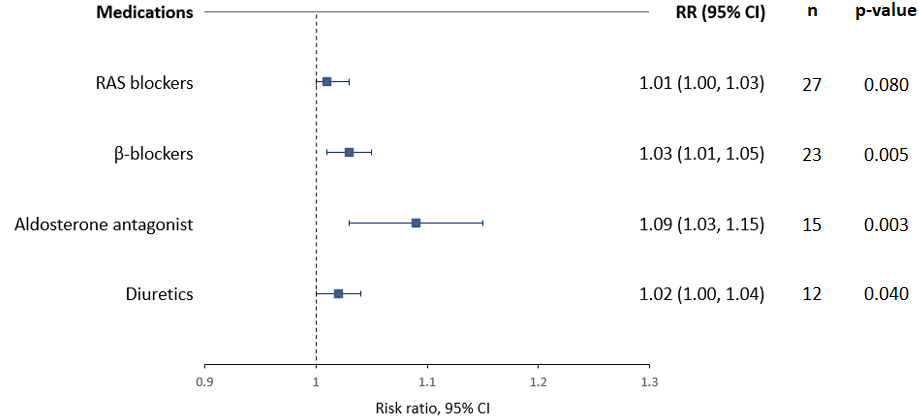


Footnotes: CI, confidence interval; n, number; RAS, renin-angiotensin system

**Table S1.** Search strategy for meta-analysis and systematic review

| 1. “Structured” OR “tailored” OR “shared” OR “multidisciplinary” OR “interdisciplinary” OR “multicomponent” OR “multifaceted” OR “integrated” OR “coordinate*” OR “collaborat*” OR “Quality” 2. “care” OR “treat*” OR “service*” OR “team*” OR “plan*” 3. “Quality improvement” OR “Quality assurance” OR “Education” OR “self-management” OR “Peer*” OR “nurse*” OR “dietitian*” OR “pharmacist*” OR “Tele*” OR “electronic health” OR “e-health” OR “Ehealth” OR “digital*” 4. “heart failure” OR “HFrEF” OR “HFpEF” OR “ischemic heart disease” OR “cardiac failure” OR “coronary artery disease” OR “myocardia*” OR “atherosclerosis” OR “arteriosclerosis” 5. (1 AND 2) AND 4 AND 5 |
| --- |
| ((“Structured” OR “tailored” OR “shared” OR “multidisciplinary” OR “interdisciplinary” OR “multicomponent” OR “multifaceted” OR “integrated” OR “coordinate*” OR “collaborat*” OR “Quality”) AND (“care” OR “treat*” OR “service*” OR “team*” OR “plan*”)) AND (“Quality improvement” OR “Quality assurance” OR “Education” OR “self-management” OR “Peer*” OR “nurse*” OR “dietitian*” OR “pharmacist*” OR “Tele*” OR “electronic health” OR “e-health” OR “Ehealth” OR “digital*”) AND (“heart failure” OR “HFrEF” OR “HFpEF” OR “ischemic heart disease” OR “cardiac failure” OR “coronary artery disease” OR “myocardia*” OR “atherosclerosis” OR “arteriosclerosis”) |

**Table S2.** Definitions of quality improvement strategies in chronic heart failure from previous meta-analysis (1)

| Category | Definition |
| --- | --- |
| **Healthcare system** | |
| Case management | - Coordination of routine management of patients in collaboration with, or supplementary to the primary care practitioners by:  1. a person: healthcare providers, trained peers or community health workers, social workers. 2. presence of a multidisciplinary team. |
| Team change | - Changes to the structure/organization of the primary healthcare team, with the presence of any of the following:  1. adding a team member or collaborative care, e.g., routine visits from heart failure specialist nurses, pharmacists, nutritionists, and psychologists. 2. use of a multidisciplinary team, e.g., medicine, nursing, pharmacy, nutrition, psychology. 3. expansion or revision of professional roles, e.g., prescription autonomy to nurses or pharmacists.  - Studies with “case management” could qualify as “team changes” if at least two of the above conditions were met. |
| Electronic patient registry | - Design of a new electronic medical record or tracking system, or improvement in the pre-existing electronic system during the study period. |
| Facilitated relay of patient’s information to clinicians | - Health information exchange between patients and healthcare providers by methods other than the traditional medical records, e.g., personal reports, trained peers or community health workers, structured self-monitoring of blood pressure/electrocardiogram/dietary/exercise diaries, electronic transmission of self-care data, e.g., heart rate, blood pressure, pulse oximetry and body weight. - Included access to out-of-office consultation to primary care practitioners and patients, feedback meetings with trained peers or community health workers with subsequent changes in patient’s management plan and improved referral system. - This information must get to someone with prescribing and ordering ability. |
| Using electronic health (eHealth) with support from heart failure team | - Involved applying software or electronic applications to promote better heart failure care, e.g., telemedicine/telemonitoring (remote monitoring of physiologic data with broadband, digital, wireless, satellite or Bluetooth transmission to a monitoring center), mobile health (m-Health), e-Learning (smartphone apps, short messaging service, videotape, automated educational messages, multimedia use, emails, personal digital assistant). - Included enhanced use of electronic databases, i.e., integration, analysis, interpretation, and communication of the information to healthcare team and patients (e.g., electronic patient’s report card, risk assessment analysis). |
| Continuous quality improvement | - An iterative process for testing the effects, assessing quality problems, providing solutions, and reassessing the need for further action (plan-do-study-act cycles, quality assurance). - Checking on intervention fidelity or feedback on intervention delivery by trained peers or community health workers with solutions provided to enhance patient’s care. |
| **Healthcare providers (clinicians, physicians, general practitioners, pharmacists, etc.)** | |
| Audit and feedback | - Benchmarking reports on the clinical performance of healthcare providers or practices on care processes. - The information may be obtained from the medical records or online databases. |
| Clinician education | - Continuous provision of up-to-date heart failure care management and guidelines to all healthcare providers, e.g., conferences/workshops, distribution of educational materials (written, video etc.), and academic detailing. - Education related to the workflow of the implementation of heart failure care model was not categorized as clinician education. |
| Clinician reminders | - Electronic system (email/phone/short message service) prompts to healthcare providers on patient-specific information (biomedical data or care processes), including ad-hoc clinician reminders. - It is sub-classified as decision support with the provision of treatment algorithms and/or protocols to healthcare providers. |
| Financial incentive (pay for performance) | - Any financial incentives (positive/negative) related to healthcare performance given to healthcare providers and patients. - Changes in reimbursement for pate as a token of achievement after participation in the program (e.g., capitation, prospective payment, or a shift from fee-for-service to salary pay structure), lower annual fee in case of treatment targets attainment. - Excluded transport reimbursement, honorarium, gift cards, or stipend to patients, healthcare providers or trained peer/community health workers for any study procedures unless they contribute to treatment targets attainment. |
| **Patients** | |
| Patient’s education | - Delivery of educational lesson/seminar/individual/group sessions on heart failure related topics by health personnel or trained peer/community health workers to the patients. - To promote better understanding of heart failure and related topics, as well as adoption of positive attitudes towards patients’ active participation in care improvement of their disease. - Distribution of printed/electronic educational materials or patient’s report card. |
| Promotion of self-management | - Provision of patient’s report card, diaries, or equipment (e.g., weight balance, pulse oximetry, electrocardiogram recorder, blood pressure machine etc.). - Access to resources only after attending education programs (e.g., online platform for transmission of self-care records to healthcare providers or to the monitoring center, facilitated adjustments of medication dose, ~~o~~n-site grocery shopping, personalized goal setting and action plan). - Involvement of trained peers or community health workers. |
| Patient reminder system | - Any effort (e.g., in person, postal mail, live/automated phone calls, mobile texts, web/emails) to remind patients about appointments or important self-care aspects. - If case management was included, patient’s reminders needed to be explicit and an extra task to the normal case management. |

**Table S3.** Key characteristics of the 105 included randomised clinical trials

| **Author, year (ref.)** | **Country** | **Study design** | **Study setting** | **Study population** | **Mean age (sd) in years** | **Male (%)** | **NYHA class** | **EF, mean (sd) in %** | **Overall study personnel(s) involved** | **Intervention/Comparator group (duration)** | **Follow-up in months** | **Relevant outcome measures** |
| --- | --- | --- | --- | --- | --- | --- | --- | --- | --- | --- | --- | --- |
| Agvall, 2014 (2) | Sweden | Open-label RCT | 5 primary healthcare centres | 160 (systolic HF)  IG: 79  CG:81 | 75 (7.8) | 68.8 | I-III | <50% | Nurse, general practitioner (GP) | Patient education intervention (6 months):   - Validated computer-based information program and HF education. - HF nurse telephone support contact + home visit post-discharge.   Control group: usual care according to local guidelines | 12 | All-cause mortality, change in medication |
| Angermann, 2012 (3) | Germany | Open, 2-armed, multicentre RCT | 9 hospitals in Bavaria and Baden-Württemberg | 715 (systolic HF)  HNC: 352  CG: 363 | 68.6 (12.2) | 60 | III-IV | 30 (8) | Specialist nurse, physicians, multidisciplinary team (MDT) | HeartNetCare-HF (HNC) program (6 months):   - Inpatient patient education + telephone-based monitoring post-discharge🡪 weekly or fortnightly for NYHA class III-IV; monthly for NYHA class I-II)   Control group: usual care   - Standard post-discharge planning, fixed appointments. | 6 | All-cause mortality, rehospitalization |
| Antonicelli, 2016 (4) | Italy | Multicentre RCT | 6 Italian Cardiologic Rehabilitation Centres | 343 (elderly + stable CHF, 43% > 75 years)  IG: 170  CG: 173 | 76.90 (5.67) | 56.9 | II-IV | Mean 48.4% | physiotherapist, nurse-tutor, cardiologist | Training care group: Exercise training (6 months):   - 1^st^ phase: 3 trainings weekly, 50-min classes (3 months); 2^nd^ phase: scheduled telephone call (3 months)   Control group: usual care   - Outpatient appointment within 2 weeks post-discharge, structure follow-up | 6 | All-cause hospitalization |
| Arjunan, 2020 (5) | India | RCT | Tertiary care hospital | 200 (hospitalised HF patients)  IG: 100  CG:100 | 59.57 (11.16) | 78.5 | NA | NA | Nurse, physician, physiotherapist | Nurse-led cardiac rehabilitation program (3 months):   - 3 sessions of structured patient education during hospitalisation. - Tele-monitoring: every 2 weeks over the 3m post-test period   Control group: routine care | 3 | Mortality |
| Austin, 2005 (6) | UK | RCT | Acute medical unit, medical & cardiology outpatient | 200 (>60y, chronic HF)  IG: 100  Comparator group: 100 | 71.9 (6.3) vs 71.8 (6.8) | 66 | II-III | ≤40 | Cardiologist, specialist nurse, exercise instructor | Intervention group (24 weeks):   - Cardiac rehabilitation programme twice weekly for 8 weeks. - 8 weekly clinical status monitoring in outpatient cardiology clinic + self-management education - Additional education input with weekly group sessions on variety of essential topics from MDT + 16 weekly 1-h exercise sessions.   Clinic based care group:   - 8 weekly clinical status monitoring in outpatient cardiology clinic + self-management education. | 6 | Hospital admission, change in medication |
| Bakitas, 2020 (7) | USA | Single-blind RCT | Academic tertiary medical centre (South-eastern US) and Veterans Affairs medical centre | 415 (HF patients, ≥50 years, 55% African American)  IG: 208  CG: 207 | 63.8 (8.5) | 53.3 | III-IV | IG vs CG: 40.9 (16.4) vs 41.2 (15.2) | Palliative Care Nurse Coach (PNC), advanced HF clinicians, study investigators | ENABLE CHF-PC (48 weeks):   - In-person comprehensive Palliative Care Team (PCT) Consultation. - Phone-based 6-session patient and 4-session caregiver curriculum + monthly phone-based supportive care for 48 weeks/ patient death.   Control group: usual care   - Outpatient management according to national HF guidelines. - HF education was provided verbally and through printed materials based on perceived patient needs. | 11 | ED visit, mortality |
| Barker, 2012 (8) | Australia | RCT | Public teaching hospital | 120 (CHF, hospitalised for at least 48h)  IG: 64  CG:56 | IG vs CG: 73.02 (10.11) vs 72.02 (10.12) | 45.8 | I-IV | 32.64 (19.70) vs 47 (20.08) | Pharmacist | Patient education intervention (6 months):   - Usual care + post-discharge home medication review for intervention group during home visit. - Patients received direct pharmacy advice.   Control group: usual care   - Home visit by pharmacist + patients’ advice upon request. | 6 | Mortality, hospital readmissions |
| Bekelman, 2015 (9) | USA | Multi-site RCT | 4 VA Medical Centres (Denver, Palo Alto, Richmond, and Seattle) and affiliated clinics | 384 (VA patients with CHF, white race (81.1%))  IG: 187  CG: 197 | IG vs CG: 67.3 (9.6) vs 67.9 (10.6) | 96.6 | I-IV | NA | MDT: nurse coordinator, cardiologist, psychiatrist, primary care physician | Patient-Cantered Disease Management (PCDM) intervention (12 months):   - - MDT collaborative HF disease management   - Telemonitoring with patient self-care support   - Screening and treatment for comorbid depression: 11 sessions of behavioural activation and antidepressant management, depression educational video   Control group: usual care   - Regular care + regular telehealth with no involvement of study collaborative care team | 12 | Hospitalization, mortality |
| Bekelman, 2018 (10) | USA | Single-blind, 2-arm, multisite RCT | Outpatient centres | 314 (symptomatic HF, 72% white race)  IG: 157  CG: 157 | 64.5 (10.9) vs 66.5 (11.8) | 78.7 | I-IV | NA | Nurse, social worker, MDT (primary care clinician, cardiologist, palliative care physician) | Collaborative Care to Alleviate Symptoms and Adjust to Illness (CASE) intervention:   - - 3 components:   - 6 nurse intervention follow-up assessments by telephone (1-2 / month)   - Social worker: structured telephone-based psychosocial intervention   - MDT reviewed patients’ care and provided tests orders and medications as needed   - Weekly meeting and discussion between nurse, social worker and MDT based on patients’ EMR   Control group: usual care | 12 | Hospitalization, mortality |
| Berger, 2010 (11) | Austria | 3-arm, prospective, randomized pilot study | 8 Viennese hospitals | 278 (clinical signs of cardiac decompensation)  MC: 96  BM: 92  CG: 90 | MC: 73 (11)  BM: 70 (12)  UC: 71 (13) | 64.7 | III-IV | <40% | CHF nurse, primary care physician, HF specialists of the research team | Multidisciplinary care (12 months):   - - Home visits and telephone contact visits + pre-scheduled consultations from the CHF specialist   - Patient and caregiver education + enhancement of self-management   N-terminal pro–B-type natriuretic peptide-guided, intensive patient management (BM):   - - Discharge at NT-proBNP level >2,200 pg/ml; ambulatory visits at a CHF specialist at least every 2 weeks   - NT-proBNP level <2,200 pg/ml🡪MC follow-up   Control group: usual care | 12 | All-cause mortality, change in medication |
| Bernocchi, 2018 (12) | Italy | Multicentre, open label RCT | Hospitals | 112 (combined COPD and CHF)  IG: 56  CG: 56 | 70 (9) | 82.1 | II-IV | 44.5 (12.4) vs 43.3 (13.2) | Nurse tutor, nurse, physiotherapist tutor | Home-based rehabilitation (4 months):   - - Patient education + remote monitoring of cardiorespiratory parameters + weekly phone-calls by the nurse   - Exercise programme with weekly monitoring by the physiotherapist   Control group:   - - Standard care programme, patients were given an educational session and were invited to practice daily physical activity as preferred. | 6 | All-cause mortality |
| Blum, 2014 (13) | USA | RCT | University of Maryland Medical Centre, Baltimore Veterans Administration Medical Centre, and private cardiology practices | 206  (HF with systolic or diastolic dysfunction)  IG: 104  CG: 102 | IG vs CG: 73 (8) vs 72 (10) | 70 vs 72 | II-IV | ≤ 40 | Research nurse coordinator, nurse | Intervention group (12 months):   - - Remote monitoring of daily vital signs using the Philips Electronics E-care System.   - Abnormal clinical parameters were flagged for the nurse practitioners for further investigation and action.   Control group: usual care | Mean: 802 ± 430 days; median: 823 days; range (33-1614) | Hospitalizations |
| Bocchi, 2008 (14) | Brazil | Randomized, prospective, parallel trial | HF clinics, university medical school | 350 (irreversible chronic HF ≥ 6 months)  IG: 233  CG: 117 | IG vs CG: 50 (19) vs 52(11) | 71.2 vs 64.1 | I-IV | ≤ 45 | Nurses, cardiologists, pharmacists, social workers, dietitians, dentists, and psychologists | Disease Program Management (6 months):   - - Patients and caregivers’ education; medication management based on guidelines   - Remote monitoring + face-to-face individual/group communication and telephone in-person communication   Control group: usual care | 2.47±1.75 years | Hospitalization, mortality |
| Bouvy, 2003 (15) | Netherlands | RCT | Cardiology outpatient clinic, hospitals | 152 (HF)  IG: 74  CG: 78 | IG vs CG: 69.1 (10.2) vs 70.2 (11.2) | 53(72)  vs 47(60) | II-III | NA | Pharmacist, GP | Pharmacist-led intervention (6 months):   - - Monthly consultations on medication compliance in patients with HF + structured interview with patients.   Control group: usual care | 6 | Hospital readmission, mortality |
| Boyne, 2012 (16) | Netherlands | Multicentre RCT | 3 hospitals in the South of the Netherlands | 382 (HF, mean, 61% with LVEF ≤0.45%)  IG: 197  CG: 185 | Median (Interquartile range (IQR))71.5 (32–93) | 59 | II-IV | 36 (28–48) | HF nurse, nurse assistant | Telemonitoring intervention:   - - Health Buddy device- daily pre-set dialogues communication with patients, pre-set questions for patients to answer.   - Risk stratification and management based on risk classification.   Control group: usual care | 12 | Readmission, all-cause mortality |
| Chen, 2019 (17) | China | Non-blinded RCT | Cardiology department of West China Hospital | 767 (HF, 68.4% with NYHA class III or IV)  STS: 255  SMS: 252  UC: 260 | 61(15) | 56.5 | III-IV | <50 | Research nurses, HF specialists, senior nursing specialists | STS group:   - - Structured phone call from research nurses after discharge + inpatient education + standard care.   SMS group:   - - Patients and caregivers received standardized messages from text messaging platform (educational SMS and reminder SMS).   Control group: usual standard care   - - Inpatient education + standard care   - Patients were not contacted in any form after discharge. | 6 | Readmissions, mortality |
| Chew, 2021 (18) | Singapore | Two-arm parallel RCT | National Heart Centre Singapore | 104 (HF, 77.1% non-smokers)  IG: 72  CG:72 | 60.6 (12.5) | 79.2 | I-III | 33.7 (12.5) | Cardiologist, post-graduate registered nurse, psychologist | Self-regulation intervention (3 months):   - 1 face-to-face session, print booklet and 3 reinforcement telephone follow-ups   Control group: usual care | 12 | Hospitalisation, ED visit, mortality |
| Cockayne, 2014 (19) | UK | Pragmatic, open parallel group RCT | HF diagnostic clinics and primary care in 7 centres in the Birmingham &Darlington areas | 260 (symptomatic HF)  IG: 95  CG: 165 | 70.60 (11.49) | 72.3 | I-IV | NA | HF nurses | Nurse-facilitated self-management programme (The Heart Failure Plan):   - - Nurse facilitated CBT self-management program + 6 structures one-to-one education sessions.   Control group: usual care   - - Same self-management manual was given without individual coaching session. | 12 | Hospital admissions, all-cause mortality |
| Comín-Colet, 2016 (20) | Spain | Open label RCT | Hospital del Mar, Barcelona | 178 (HF, 25% frail patients)  IG: 81  CG: 97 | 74 (11) | 69 | I-IV | 47(16) | Case manager, primary care doctor, nurses, primary care cardiologist | HF programme +Telemedicine:   - - Daily signs and symptoms telemonitoring using Tele-HealthCare platform.   - Structured follow-up (video or audio conference).   - HF nurses reviewed alarms and alerts from system.   HF programme only:   - - Structured follow-up on the basis of face-to-face encounters.. | 6 | Hospital readmission, mortality |
| Dalal, 2019 (21) | UK | Multicentre RCT | Hospitals (Birmingham, Cornwall, Gwent and York) | 216 (systolic HF)  IG: 107  CG: 109 | IG vs CG: 69.7 (10.9) vs 69.9 (11) | 78 | I-IV | 34 | Community HF specialist nurse, physiotherapists, family doctor, cardiologist | REACH-HF intervention (12 weeks):   - - Usual care + facilitated self-care and home-based cardiac rehabilitation program delivered via a mixture of face-to-face and telephone contacts.   - Comprehensive intervention with 4 core elements:   - Structured exercise programme   - Patient ‘Progress Tracker’   - ‘Family and Friends Resource’   - Facilitation by cardiac nurses/physiotherapists   Control group: usual care | 12 | Mortality, hospitalization |
| Dar, 2009 (22) | UK | Multicentre RCT | 3 acute hospitals | 182 (HF, 20% of South Asian, 45% aged ≥ 75 years)  IG: 91  CG: 91 | 72 (19) | 66 | II-IV | ≥40 | Study nurse, specialist nurse, physician, cardiologist | Telemonitoring group (TM):   - - Usual care plus self-monitoring (Honeywell HomMed™).   - Patient data were reviewed by a HF nurse.   Control group: usual care | 6 | Hospitalization, ED visits |
| Davidson, 2010 (23) | Australia | RCT | Teaching Hospital Facility In Metropolitan | 105 (HF)  IG: 53  CG: 52 | 71.6 vs 73.9 (mean only) | 64.2 vs 59.6 | I-IV | NA | Cardiac rehabilitation coordinator, cardiologist/GP, MDT: nurse, pharmacist, physiotherapist, dietitian, occupational therapist | Multidisciplinary CR program (12 weeks)   - - Weekly, home-based individualized exercise program by MDT: patient self-management and treatment adherence monitoring.   - Usual follow-up at the outpatient clinic.   Control group:   - - One information session provided by the cardiac rehabilitation coordinator via phone call.   - Usual care + follow-up | 12 | Rehospitalization, all-cause mortality |
| de la Porte, 2007 (24) | Netherlands | RCT | 2 regional teaching hospitals | 240 (HF)  IG: 118  CG: 122 | IG vs CG: 70 (10) vs 71 (10) | 66 vs 79 | III-IV | 31 | Cardiologists, dietitian, HF physician and cardiovascular nurse | Intervention group (12 months):   - - Usual care + 9 scheduled patient contacts: by telephone/ by a visit to an intensive physician‐and‐nurse‐directed HF outpatient clinic   - Comprehensive patient education, optimization of treatment, easy access to the clinic, exercise and rest recommendations, advice for symptom monitoring were provided to the patients   Control group: usual care | 12 | All-cause mortality, change in medication |
| DeBusk, 2004 (25) | USA | RCT | 5 northern California hospitals | 462 (hospitalised HF patients)  IG: 228  CG: 234 | 72 (11) | 48 vs 54 | I-IV | NA | Nurse, physician, nurse-care managers | Intervention group:   - - Usual care + telephone-mediated nurse care management   - Structured telephone surveillance, coordination of patients’ care with primary care physicians.   Control group: usual care | 12 | Rehospitalization, ED visits, mortality |
| Del Sindaco, 2007 (26) | Italy | RCT | 2 hospital HF clinics | 173 (elderly HF patients)  IG: 86  CG: 87 | 77 (6) | 52 | II-IV | 33.5 (11) vs 32.5 (10) | Cardiologist, geriatrics, specialised nurses, primary care physician | Disease management program:   - - Discharge planning, patient education, therapy optimisation, improved communication, early attention to signs and symptoms + intensive follow-up   Control group: usual care | 24 | Mortality, hospital admissions |
| Dendale, 2012 (27) | Belgium | Multicentre RCT | 7 hospitals throughout Belgium | 160 (chronic HF)  IG: 80  CG: 80 | 76 (10) | 65 | NA | 35 (15) | GP, HF nurse, HF clinic team | TM group (6 months):   - - Usual care + daily measurement of vital signs; results were forwarded to the central computer to intervene when pre‐defined limits were exceeded   - Email alert for GP   Control group: usual care   - - Subjects and close relatives received standard education course before discharge + standard HF care | 6 | All-cause mortality, hospitalizations, change in medication |
| Dessie, 2021 (28) | Ethiopia | Two-arm, parallel, clustered-RCT | Debre Markos Referral Hospital, Felge Hiwot Referral Hospital | 219 (Chronic HF)  IG: 88  CG: 98 | Median (IQR) IG vs CG: 50 (30-60) vs 37.5 (25-50.75) | 41.4 | I-IV | NA | Project team (nurses, physicians, nutritionists, health educators, and physiotherapists) | Self-care education intervention group (12 months):   - - Usual care + intensive four-day educational training, one-day follow-up sessions every 4 months.   - Patients received self-care education.   Control group: usual care   - - Basic discharge instructions | 12 | Hospitalization |
| DeWalt, 2006 (29) | USA | RCT | University of North Carolina (UNC) General Internal Medicine Practice | 123 (HF, on furosemide drug, 41% had inadequate literacy)  IG: 59  CG: 64 | IG vs CG: 63 (9) vs 62 (11) | 58 vs 41 | II-IV | NA | RA, educator, primary care physician | Intervention group (12 months):   - - Patient education on diuretic dose self-adjustment, self-emphasizing daily weight measurement, symptom recognition and response + self-management reinforcement and feedback   - Scheduled follow-up phone calls regularly.   Control group: usual care  General HF education pamphlet | 12 | All-cause mortality, cardiac hospitalizations |
| DeWalt, 2012 (30) | USA | Multisite randomized trial | 4 academic centres in North Carolina | 605 (HF patients)  Single session: 302  Multisession: 303 | 60.7 (13.1) | 52 | II-IV | <0.45 | RA, educators, investigators | Single session:   - - Patients received a 40-minute in-person, literacy-sensitive training.   Multisession:   - - Similar initial training as above + ongoing telephone-based support. | 12 | All-cause mortality |
| Doughty, 2002 (31) | New Zealand | Cluster RCT | Hospital-based HF clinic | 197 (HF)  IG: 100  CG: 97 | IG vs CG: 72.5 (11.6) vs 73.5 (10) | 60.4 | III-IV | 30.6 (12.7) vs 33.8 (12.7) | Study nurse, cardiologist, GP | Intervention group:   - - Clinical review early after discharge + individual and group education sessions + usual care.   - Patients were given a personal diary to record medication and body weight.   Control group: usual care | 12 | All-cause mortality, change in medication |
| Du, 2018 (32) | Australia | Multicentre RCT | 3 academic hospitals in Sydney | 132 (symptomatic HF patients)  IG: 67  CG: 65 | 60 (15) | 78.7 | II-III | 32.6 (12.5) | Nurse, researcher, cardiologist | Home-Heart-Walk group (6 months)   - - Usual care + weekly HHW   - Patient education and self-monitoring: record weekly result in a HHW program booklet.   - Research nurse reminded participants of their weekly HHW.   Control group: usual care   - - Bedside education from nurse before discharge.   - Regular appointments with cardiologist. | 6 | All-cause mortality |
| Dunbar, 2015 (33) | USA | RCT | 1 of 4 large urban tertiary care hospitals | 134 (HF and DM, 69% minority)  IG: 70  CG: 64 | 57.4 (11) | 66 | II-III | 33.3 (17.6) | Research nurse, clinicians | Intervention group (6 months):   - - Education/counselling on combined HF and DM self-care (diet, medications, self-monitoring, symptoms, and PA) with follow up home visit and phone counselling.   Control group:   - - Standard HF and DM educational brochures with follow up phone contact. | 6 | All-cause mortality |
| Ekman, 1998 (34) | Sweden | RCT | Sahlgrenska University  Hospital | 158 (Chronic HF)  IG: 79  CG: 79 | 80.3 (6.8) | 57.6 | III-IV | 0.43 (0.18) vs 0.38 (0.15) | Nurse, study doctor | Structured care group:   - - Nurse-monitored, outpatient-care program aiming at symptom management.   - Nurse regularly contacted the patients by telephone to follow-up the discussions during the clinic visits.   Control group: usual care | 6 | Hospital readmission, all-cause mortality |
| Ekman, 2003 (35) | Sweden | RCT | Sahlgrenska University Hospital | 145 (CHF, aged >65 years)  IG: 70  CG: 75 | IG vs CG: 80.4 (7.3) vs 79.3 (6.5) | 61.4 | III-IV | IG vs CG: 41 vs 37 | Nurse, physicians | Nurse-monitored structured care program:   - - Education about CHF and its treatment + advice on weight control.   - Up-titration of enalapril to a target dose twice a day.   Control group: standard care | 6 | Change in medication |
| Elpida, 2021 (36) | Greece | Single-centred RCT | General hospital | 122 (chronic HF, 62.3% with reduced ejection fraction)  IG: 61  CG: 61 | 67.1 (12.3) | 83.6 | I-IV | IG vs CG:  36.8 (9.9) vs 36.4 (9.9) | HF specialists, cardiology specialists, physiotherapists, specialist HF nurse, occupational therapists, psychologists, social workers | Constructive teaching method:   - - 5 phases: orientation, elicitation, restructuring, application, and review.   Control group:   - - Standard health care   - Patients were excluded from restructuring and application phase. | 6 | Unplanned hospital readmission, mortality |
| Freedland, 2015 (37) | USA | RCT | Washington University Medical Centre in St. Louis | 158 (HF patients with comorbid major depression)  CBT: 79  CG: 79 | 55.8 (11.2) | 53.8 | I-III | 38.9 (15.5) | 2 masters-level and 2 doctoral-level therapists, cardiac nurse | Cognitive Behavioural Therapy (CBT) (6 months):   - - Usual care + structure HF education program + CBT sessions.   - Individualized treatment, progress monitoring and adjustment of treatment.   - Weekly supervision meetings   Control group: enhanced usual care   - - Structure HF education program | 12 | Hospitalization |
| Gattis, 1999 (38) | USA | RCT | Outpatients University Medical Centre | 181 (HF with left ventricular dysfunction)  IG: 90  CG: 91 | IG vs CG: 68.84 (12.6) vs 64.22 (12.94) | 69 vs 67 | I-IV | <45 | Clinical pharmacist, physician, nurse practitioner | Intervention group (6 months):   - - Clinical pharmacist evaluation: medication evaluation, therapeutic recommendations to the attending physician   - Patient education + follow-up telemonitoring   Control group: usual care | 6 | Mortality, change in medication |
| GESICA, 2005 (39) | Argentina | Multicentre RCT | 51 centres in Argentina (public and private hospitals and ambulatory settings). | 1518 (outpatients with stable chronic HF)  IG: 760  CG: 758 | 65 (13.3) | 72.6 vs 68.9 | III-IV | NA | Nurse, attending cardiologists | Telephone intervention group (6 months):   - - Usual care + education, counselling, and monitoring by nurses through telephone follow-up   Control group: usual care | 6 | All-cause mortality, hospital admission, change in medication |
| Güder, 2015 (40) | Germany | RCT | Hospital | 706 (decompensated HF)  HNC: 343  CG: 363 | 66.2 (12.5) | 73.2 | III-IV | 29.6 (7.1) | HF-specialist nurse, physician, cardiologist | HeartNetCare-HF (HNC) program (18 months):   - - Nurse-coordinated HNC program with self-management training during hospitalization   - Patient education on self-monitoring + telephone follow-up at post-discharge   Control group: usual care | 18 | Change in medication, all-cause mortality |
| Harrison, 2002 (41) | Canada | RCT | 2 general medical units of a large urban teaching hospital | 192 (CHF)  IG: 92  CG: 100 | 87 (45) | 55 | NA | NA | Home-care coordinator, nurses, physician | Transitional care group (12 weeks):   - - Standard discharge planning, comprehensive program, supportive care for self-management were provided to the patients.   Control group: usual care | 3 | Hospital readmission, ED visits, mortality |
| Heisler, 2013 (42) | USA | RCT | Community-based teaching hospital | 267 (diastolic or systolic HF)  RPS: 136  Comparator group: 131 | 69.1(12.1) | 49 | NA | NA | HF nurse practitioner, research associate | Reciprocal Peer Support (RPS) (6 months):   - - Nurse practitioner-led goal setting group session + 3 sessions of 1.5-hour group sessions facilitated by nurse practitioner for sharing and progress update.   - Patient HF self-management, peer communication skills training and interaction between participants weekly using a telephone platform.   Comparator group:   - - Nurse care management + one 1.5-hour HF self-management session   - Patients were given HF educational materials and information on care management services. | 12 | Composite of rehospitalization and all-cause mortality |
| Jaarsma, 2000 (43) | Netherlands | RCT | University Hospital in Maastricht | 186 (HF)  IG: 89  CG: 97 | 72 (9) | 58 | III-IV | NA | Study nurse, cardiologist, physician | Intervention group (9 months):   - - Systematic education and support by a nurse in the hospital and at home (average 4 hospital visits, 1 telephone call and 1 home visit)   Control group: routine care | 9 | All-cause mortality |
| Jiménez-Marrero,2020 (44) | Spain | Single centre, open label RCT | Bellvitge University Hospital | 178 (28% frail patients, after discharge for an acute HF decompensation)  IG: 81  CG: 97 | 77 (10) | 53 | III-IV | 58 | MDT: CHF physician specialist, nurse | Telemedicine group (6 months):   - - Structured follow-up (remote/on-site; using video conference/audio conference)   - Health education, pharmacological up-titration intervention, and self-monitoring of patients’ bio-measures daily and record via a software which will be transferred to the HF unit🡪 clinician alert system   Control group: usual care   - - On-site, face-to-face, structured follow-up   - Health education and pharmacological up-titration intervention + patients self-monitoring of their bio-measures daily and contact the nurses in the event of any abnormality | 6 | Composite of hospitalizations and mortality |
| Jolly, 2009 (45) | UK | RCT | Specialist HF services centres | 169 (CHF)  IG: 84  CG: 85 | IG vs CG: 65.9 (12.5) vs 70.0 (12.5) | 74.5 | I-III | ≤40 | Specialist nurse | Intervention group (6 months):   - - Home-based walking and resistance exercise programme + specialist nurse care.   - 3 supervised exercise sessions (individualized exercise program) + self-management and monitoring, titration of beta-blocker therapy.   Control group: Specialist nurse alone   - - Advice about self-management and monitoring, titration of beta-blocker therapy | 12 | Composite of death, hospital admission |
| Kalter-Leibovici, 2017 (46) | Israel | Multicentre, open-label RCT | Community heart failure centres, hospital | 1360 (HF)  IG: 682  CG: 678 | 70.7 (11.3) | 72.5 | I-IV | >50; <50 | MDT: nurse, dietitians, social workers,  primary care practitioners and consultant cardiologists | Disease management group:   - - Coordination of care, patient education, monitoring disease symptoms and patient adherence to medication regimen, titration of drug therapy, and home tele-monitoring of vital signs.   Control group: usual care | 2.67 (1.22) | Hospital admission, all-cause death |
| Kasper, 2002 (47) | USA | RCT | The Johns Hopkins Hospital and The Johns Hopkins Bayview Medical Center | 200 (chronic HF)  IG: 102  CG: 98 | IG vs CG: 60.2 (13.8) vs 63.7 (15.0) | 60.5 | II-III | <45% | Cardiologist, CHF nurse, telephone nurse coordinator, primary physician | Multidisciplinary outpatient management program (6 months)   - - Regular telephone follow-up tailored therapeutic plan, and medications adjustment.   - Clinician reminders of abnormal laboratory values.   - Patients were supplied with a pill sorter, a list of correct medications, a list of dietary and physical activity recommendations, a contact number available 24 h/day and patient education material   Control group: usual care | 6 | Mortality, change in medication |
| Kenealy, 2015 (48) | New Zealand | Multisite RCT | Major city hospital contributed patients with CHF/COPD (site A&B); remote primary site in a rural settlement (Site C) | Total: 171  IG: 98  CG: 73  Site A: 98 (CHF)  IG: 49  CG: 49  Site B: 48 (COPD)  Site C: 25 (mostly diabetes) | Median (IQR) 72 (62-83) vs 72 (60-77) | 66.3 | I-IV | NA | Cardiologist, HF nurse specialists, project manager | Intervention group- telecare (6 months):   - - Patient level: self-care education using website tool (e-portal ‘health hub’ telecare system) to generate regular information and to make regular self-management decisions.   - Clinicians received regular information, provide practice care based on the review of the daily feedback via telecare, give advice to patients.   Control group: usual care  Self-care education using a detailed tool on a website | 6 | All-cause mortality |
| Köberich, 2015 (49) | Germany | Single-centre, multi-site, nonblinded, RCT | University‐affiliated medical centre in southern Germany | 110 (LVEF ≤40%)  IG: 58  CG: 52 | 62 | 82.7 | II-IV | 28.2 | Principal investigator, nurse | Intervention group (12 weeks):   - - Usual care + patient education session and telemonitoring via phone calls after discharge.   Control group: usual care | 3 | All-cause mortality |
| Koehler, 2011 (50) | Germany | Open label, multicentre RCT | Outpatient clinic | 710 (stable chronic HF)  IG: 354  CG: 356 | IG vs CG: 66.9 (10.8) vs 66.9 (10.5) | 81.3 | II-III | 26. (5.7) vs 27.0 (5.9) | Physicians, nurses | Physician-led remote telemedical management:   - - Usual care + daily telemonitoring of ECG, blood pressure, and body weight   - Patients were contacted to verify measurements, to give consultation, or to institute treatment.   Control group: usual care | 24 | Composite of death and hospitalizations |
| Koehler, 2018 (51) | Germany | Multicentre RCT | Hospitals and cardiology practices | 1538 (HF, BMI 30 (6))  RPM: 765  CG: 773 | IG vs CG: 70 (11) vs 70 (10) | 69.6 | II-III | 41 (13) | Telemedicine central staff, GP, local cardiologists, certified nurses | Remote patient management (RPM) group:   - - Usual care + daily remote patient management and data transmission.   - Monthly structured telephone interviews, patient education program   - Co-operation between the telemedical centre, and the patient's GP and cardiologist.   Control group: usual care | 12 | Composite of mortality |
| Koehler, 2021 (52) | Germany | Multicentre RCT | Cardiology, internal medicine, or general medicine practices | 674 (CHF)  IG: 339  CG: 335  PHQ-9< 10  IG: 260  CG: 258 | 67 (10.8) | 81.3 | II-III | 26.93 (5.78) vs 27.02 (5.74) | HF nurse, physician, GP, cardiologist | Telemedical intervention:   - - Daily remote telemonitoring and data transmission.   - Patients were given a home emergency call system to contact the telemedical centre.   - Monthly structured telephone interviews.   Control group: usual care | Minimum 12 months, maximum 28 months | All-cause mortality |
| Kotooka, 2018 (53) | Japan | Multicentre RCT | 3 outpatient clinics, 4 provincial hospitals, 6 general hospitals in urban areas, 1 national centre, and 13 university hospitals | 181 (chronic HF)  IG: 90  CG: 91 | IG vs CG: 67.1 (12.8) vs 65.4 (15.6) | 59.1 | II–III | 40.5 (14.8) vs 39.2 (16.5) | Physicians, nurses | Home telemonitoring group:   - - Patient self-care management using monitoring devices.   - Nurses monitored patient symptoms on the secure website and contacted patients by telephone and established internet communication if necessary   Control group:   - - Usual care + discharge education provided by clinician.   - Patients were encouraged to measure body weight every day | 15 | Composite of hospitalization and death |
| Laramee, 2003 (54) | USA | RCT | Hospital | 287 (CHF, left ventricular dysfunction <40%)  IG: 141  CG: 146 | 70.7 (11.8) | 54.4 | I-IV | <40% | CHF case manager, primary care physician, cardiologist | CHF case management intervention (12 weeks):   - - Early discharge planning, patient and family CHF education, promotion of optimal CHF medications and telephone follow-up.   Control group: usual care | 3 | Mortality, composite of readmissions, change in medication |
| Liljeroos, 2015 (55) | Sweden | RCT | University hospital, county hospital and outpatient clinic | 155 (chronic HF patient-partner dyads)  IG: 71  CG: 84 | IG vs CG: 69.4 (13.6) vs 72.9 (10.1) | 75.5 | II-IV | NA | HF nurses | Nurse-led program (12 weeks):   - - Usual care + educational and psychosocial support.   - Nurse-led face-to-face counselling, computer-based program, and written materials.   Control group: usual care   - - Patients received optimized treatment according to international guidelines + verbal and written patient education | 24 | All-cause mortality, composite of readmissions |
| Lowrie, 2012 (56) | UK | Cluster RCT | Primary care (174 practices) | 2169 (HF with left ventricular systolic dysfunction)  IG: 1092  CG: 1077 | IG vs CG: 70.6 (10.3) vs 70.6 (10.1) | 70 | I-IV | NA | Primary care-based pharmacists, GP, family doctor, nurse | Pharmacist-led intervention:   - - Pharmacists training, group discussion of cases/informal discussion on therapeutics, regular telephone contact between study pharmacists and the principal investigator/other pharmacist   - Patients received appointment with pharmacist for consultation + usual care   Control group: usual care | Median 4.7 years | Composite of death and hospital admission, change in medication |
| López Cabezas, 2006 (57) | Spain | Open label, RCT | Hospital of Vix and the Municipal Hospital of Badalona | 134 (HF)  IG: 70  CG: 64 | 75 | 41.4 vs 46.9 | I-IV | 54.5 (14.4) vs 47.4 (17.3) | Pharmacist, research team | Pharmacist-led intervention group (12 months):   - - Patient received information about HF disease, drug therapy, diet education and active telephone follow-up.   Control group: standard care | 12 | Hospital readmissions, all-cause mortality |
| Mehralian, 2014 (58) | Iran | Single blind, RCT | Hospital, Shahrekord, southwest of Iran. | 110 (CHF)  IG: 55  CG: 55 | IG vs CG: 61.28 (13) vs 62.7 (10) | 54 vs 62.2 | II–IV | <45% | Nurses | Group I (control group) (6 months): usual care   - - Patients received usual education at the time of hospital discharge   Group II (intervention group):   - - Usual care + special education regards to patients’ illness by nurses who visited patients in their homes.   - Patients received simplified booklet about CHF | 6 | All-cause mortality |
| Mejhert, 2004 (59) | Sweden | RCT | Danderyd University Hospital, 28 community health centres | 208 (HF with left ventricular systolic dysfunction aged ≥60 years)  IG: 103  CG: 105 | 75.8 (7.1) | 58 | II-IV | 34 | Nurse, GP, senior cardiologist, | Intervention group (18 months):   - - Nurse monitored management program: patients received education on medication titration, reinforced compliance, and dietary advice.   - Senior cardiologist supervised the program.   - Self-management of patients   Control group: usual care | 18 | Hospitalization, mortality, change in medication |
| Murray, 2007 (60) | USA | RCT | University-affiliated, inner-city, ambulatory care practice | 314 (HF, low-income patients aged ≥50 years)  IG: 122  CG: 192 | IG vs CG: 61.4 (7.7) vs 62.6 (8.8) | 32 vs 33.9 | I-IV | NA | Pharmacist, clinic nurse, physician, technician, geriatrician, cardiologist, behavioural scientist, cognitive psychologist | Pharmacist-led intervention (9 months):   - - Interdisciplinary team supported medication management by patients who have low health literacy and limited resources   Control group: usual care | 12 | Mean hospital admission, all-cause mortality, ED visits, change in medication |
| Nahlén Bose, 2016 (61) | Sweden | RCT | HF outpatient clinic | 103 (CHF)  IG: 52  CG: 51 | IG vs CG: 72.2 (9.7) vs 69.0 (8.6) | 29(65.9) vs 36 (72.0) | II-III | <30, 30-50, >50 | cardiac nurse, psychologist | CET intervention group:   - - 7 sessions coping effectiveness training (CET)   Control group: standard health care | 12 | All-cause mortality |
| Naylor, 2004 (62) | USA | RCT | 6 Philadelphia academic and community hospitals | 239 (patients hospitalized with HF and aged ≥65 years)  IG: 118  CG: 121 | IG vs CG: 76.4 (6.9) vs 75.6 (6.5) | 43 | NA | NA | APNs, physician | Comprehensive transitional care group (3 months):   - - Standardized orientation and training program guided by MDT of heart failure experts.   - Care management strategies foundational to the Quality‐Cost Model of APN Transitional Care.   - APN implementation of an evidence‐based protocol such as 3-month APN-directed discharge planning and home follow-up etc.   Control group: standard care routine | 12 | Rehospitalization, all-cause mortality, ED visit |
| Nolan, 2021 (63) | Canada | Multi-site, parallel-group, double blind RCT | Heart function clinics of 3 tertiary care hospitals | 242 (CHF)  IG: 120  Comparator group: 122 | Median (IQR); IG vs comparator group: 60 (52-69) vs 59 (52-70) | 78 | I-III | NA | Cardiologist, research staffs | e-Counselling + usual care:   - - Interactive web pages were used to provide e-counselling messages and e-tools.   - Patient-centred e-counselling protocol was created: e-sessions on planning and initiating behaviour change, interactive self-assessment tools and trackers for daily values, dramatic vignettes to validate patient experience, and video-tape discussion among CHF patients.   e-Info + usual care:   - - Enhanced usual care with online CHF self-care education.   - CHF education included trackers for self-care behaviours. | 12 | All-cause mortality |
| Norman, 2020 (64) | USA | RCT | Bryan-LGH Hospital in Lincoln, Henry Ford Hospital | 204 (HF, aged ≥19 years, 53.4% Caucasian)  IG: 102  CG: 102 | 60.4 (11.5) | 55.4 | I-IV | 39.3 (12.1) vs 40.5 (14.0) | Nurse, exercise facility coach | HEART Camp intervention (18 months):   - - Enhanced usual care plus cognitive-behavioural intervention delivered using both group-based and individual-based strategies.   Enhanced usual care group:   - - Free membership to access exercise facility and general staff were provided to the subjects.   - Subjects were encouraged to record weekly exercise diary and wear a HR monitor during exercise. | 18 | All-cause mortality |
| Nucifora, 2006 (65) | Italy | Preliminary RCT | Internal medicine department, tertiary hospital | 200 (HF)  IG: 99  CG: 101 | 73 (8) | 62 | NA | 43 (16) vs 43 (19) | Nurses, primary care physician | Intervention group (6 months):   - - Nurse-led education program, facilitated telephone communication and follow-up visits which involved problem solving, promote patients’ self-management skills, reinforce education, compliance and adherence to treatment plan.   Control group: usual care   - - Pre-existing routine of post-discharge care | 6 | All-cause mortality, readmissions, change in medication |
| O’Connor, 2009 (66) | USA, Canada, France | Multicentred RCT | 82 centres | 2331 (Medically stable outpatients with HF and reduced EF)  IG: 1159  CG: 1172 | Median (IQR), IG vs CG: 59.2 (51.2-67.8) vs 59.3 (51.1-68.2) | 72 | II-IV | Median (IQR), IG vs CG: 24.6 (20.0-30.0) vs 24.9 (20.0-30.2) | Study investigators, training personnel | Intervention group (3 months):   - - Usual care + aerobic exercise training (36 supervised sessions followed by home-based training and periodic supervised sessions for reinforcement)   Control group: usual care   - - Standard medical therapy and patients were given detailed self-management educational materials. | Median: 30 months | Composite of mortality, composite of hospitalization |
| Oddone, 1999 (67) | USA | Multisite RCT | 9 Veterans Affairs medical centres | 443 (Hospitalized with diagnosis of CHF)  IG: 222  CG: 221 | IG vs CG: 65.1 (9.1) vs 64.0 (10.9) | 99 | I-IV | NA | Primary care nurse, physician | Intervention group (6 months):   - - Enhanced access to primary care: assignment of a primary care nurse and physician, increased telephone contact, additional outpatient visits, and patient education   Control group: usual care | 6 | Hospital readmission, mortality, change in medication |
| Olivari, 2018 (68) | Italy | RCT | 8 hospitals | 339 (discharge from hospital after acute HF in previous 3 months, age ≥ 65)  IG: 229  CG: 110 | IG vs CG: 79.6 (6.8) vs 80.9 (7.3) | 61.1 vs 65.4 | II-IV | NA | Cardiologist, CHF nurse, telephone nurse coordinator, primary physician | Remote monitoring group (12 months):   - - Daily data transmission and telemonitoring to the eHealth regional centre, clinical data were collected on Personal Health System.   - Patients’ potential alarm values were identified based on their personalised telemonitoring plans.   - Personal alarm device was given to each patient.   Usual care group:   - - Routine telecare service and personal alarm device was given to each patient.   - Routine outpatient visits/ follow-ups. | 12 | All-cause death, hospital admission, change in medication |
| Ong, 2016 (69) | USA | RCT | 6 academic medical centres in California. | 1437 (decompensated HF, ≥50 years, 22% African American)  IG: 715  CG: 722 | Median 73 | 53.8 | NA | 42.7 vs 43 | Registered nurse, study nurse, physician group, pharmacist | Intervention group (6 months):   - - Predischarge HF education, regular telephone coaching, and home telemonitoring.   - Daily telemonitoring + centralized registered nurses conducted telemonitoring reviews, protocolized actions, and telephone calls.   Usual care group:   - - Predischarge education + follow-up telephone call | 6 | Readmission, all-cause mortality |
| Ortiz-Bautista, 2019 (70) | Spain | Single centre, open-label RCT | Cardiology outpatient clinic, hospital | 127 (HF, ≥18 years)  IG: 87  CG: 40 | 75 (12) | 69 | I-IV | ≤50 | Nurse care manager, nurse | Intervention group:   - - Patients were individually scheduled with the nurse for health educational counselling and evidence-based drug treatment up-titration.   - Nurse case manager follow-up visits in an outpatient clinic   Control group: standard care | Mean follow-up: 2 years | All-cause mortality, hospital readmission, change in medication |
| Østergaard, 2018 (71) | Denmark | Multi-centre  RCT | 3 Danish HF outpatient clinics in Southern Denmark and the Capital Region | 468 (80% NYHA class II)  IG: 232  CG: 236 | 66.7 (12.4) | 74 | II-IV | 27.5 (8.7) | HF-specialised nurse, project nurse | Intervention group (6-12 weeks):   - - Conventional care plus family nursing therapeutic conversations   Control group: conventional care | 3 | All-cause mortality |
| Otsu, 2011 (72) | Japan | RCT | Hospital | 102 (outpatients with CHF)  IG: 50  CG: 52 | IG vs CG: 71.6 (9.3) vs 74.6 (8.1) | 63 | II-III | NA | MDT: cardiovascular specialist, nurse (>10 years of cardiovascular nursing experience), researcher with nursing license., and/or the family | Intervention group (6 months):   - - Standard care plus additional nurse-directed educational program   Control group: medical treatment and standard care | 12 | Hospital admission |
| Panella, 2009 (73) | Italy | Cluster RCT | 14 community hospitals | 429 (CHF)  IG: 214  CG: 215 | IG vs CG: 81.7 (8.5) vs 79.7 (8.5) | 49.5 | II-IV | NA | MDT: internal medicine physicians, cardiologists, epidemiologists, pathologists, psychologists, nurses, hospital pharmacists, social workers, support staff | Intervention group (6 months):   - - Clinical pathways: appropriate therapeutic guidelines use, new organisation and procedures, and patient education   Control group: usual care | 6 | In-hospital mortality, unscheduled readmission |
| Pekmezaris, 2019 (74) | USA | Randomized comparative trial | Nassau University Medical Center | 104 (HF, 31% Hispanic)  TSM: 46  COM: 58 | 59.9 (15.1) | 59 | I-III | NA | Bilingual research nurse coordinator, cardiologist | Telehealth self-monitoring (TSM):   - - Routine care, daily vital signs self-monitoring and weekly telehealth visits.   Comprehensive outpatient management (COM):   - - Routine care + one clinic visit within a week of discharge and weekly “check-in” phone calls with the RNC during the first month of enrolment. | 3 | ED visit, composite of hospitalizations |
| Peters-Klimm, 2009 (75) | Germany | Cluster RCT | University hospital | 168 (Chronic systolic HF)  IG: 91  CG: 77 | IG vs CG: 68.4 (10.6) vs 69 (9.5) | 69 | II-IV | ≤40 | MDT: GP, cardiologist, specialist in psychosomatic medicine | Train the trainer group (18 GPs) (7 months):   - - GPs received 4 meetings addressing clinical practice guidelines and pharmacotherapy feedback.   Control group (19 GPs): usual care   - - GPs received a single 3-hour lecture by a senior cardiologist with extensive didactic expertise based on the predefined learning targets. | 7 | Hospital admission, mortality, change in medication |
| Peters-Klimm, 2010 (76) | Germany | Exploratory patient-RCT phase II | Primary care of local university | 197 (Chronic systolic HF)  IG: 97  CG: 100 | IG vs CG: 70.4 (10.0) vs 68.9 (9.7) | 72 | I-IV | ≤45 | Trained doctor's assistant (DA), GP | Case management group:   - - DAs received theoretical and practical training before conducting regular patient monitoring for 1 year by telephone or by 3 home visits for all patients.   - DAs gave feedback to GPs and enhanced patients self-management (patient leaflet, booklets, tailored diaries, etc.)   Control group: usual care | 12 | Change in medication, hospital admission, mortality |
| Philbin, 2000 (77) | USA | RCT | 10 acute care community hospitals | 1402 (CHF, 97% Caucasian, 89% NYHA class III/IV)  IG: 762  CG: 640 | 76 (11) | 44 | I-IV | NA | Physicians, nurse leaders, health professionals | Multifaceted quality improvement intervention:   - - Health professionals education about clinical issues in HF   - Patients received videotapes of the lectures to assist in dissemination of the didactic message.   - Telemonitoring of the patient post-discharge.   Control group: usual care | 6 | Change in medication, composite of hospital readmission and mortality |
| Piette, 2015 (78) | USA | Randomized comparative effectiveness trial | VA Medical center | 331 (CHF, 77% Caucasian)  Standard mHealth: 165  MHealth+CP: 166 | 67.8 (10.2) | 99.4 | II-III | <40 | Primary care physicians, cardiologists, HF nurse care managers | Standard mHealth intervention (12 months):   - - Tailored self-management advice + weekly interactive voice response calls.   - Clinical team received structured fax alerts regarding serious health concerns.   mHealth+Care Partner (CP):   - - Same as standard mHealth care + automated emails sent to patients CarePartner after each interactive voice response call with feedback and suggestions for how the CarePartner could support disease care. | 12 | All-cause mortality |
| Powell, 2010 (79) | USA | Single-centre, multi-hospital RCT | 10 hospitals located throughout Chicago metropolitan area | 902 (HF, LVEF ≤40%)  IG: 451  CG: 451 | 63.6 (13.5) | 52.7 | II-III | NA | Treatment teams, study investigators | Self-management intervention (12 months):   - - Provision of HF education information and self-management for patients.   - 18 contacts and 18 HF educational tip sheets (self-management skills, problem solving, environmental restructuring, elicitation of support from family and cognitive restructuring).   Enhanced education control group:   - - Patients were given HF educational materials by mail + a telephone called to ensure receipt, check comprehension and answer questions about the information. | 12 | Mortality and composite of hospitalization |
| Rahimi, 2020 (80) | UK | 2-armed partially blinded parallel RCT | 7 UK hospital sites | 202 (HF)  IG: 101  CG: 101 | IG vs CG: 72.8 (11.1) vs 70.4 (11.9) | 72 | I-IV | Median: 37% | IT-supported specialist, HF nurses, central clinical staff, GP | Supported medical management (intervention):   - - Active self-monitoring and IT-supported specialist management with collection of home monitoring data, blood tests, patient-reported outcomes and clinical events.   - Physicians received advice on blood investigations and pharmacological treatment from a central specialist HF team.   Enhanced self-management (control):   - - Same monitoring system as intervention group but with no central medical management support.   - Self-monitoring with collection of home monitoring data, blood tests, patient-reported outcomes and clinical events | 6 | Change in BP, hospital admission, mortality |
| Rich, 1995 (81) | USA | RCT | Medical wards of Jewish Hospital at Washington University Medical Center | 282 (CHF)  IG: 142  CG: 140 | IG vs CG: 80.1 (5.9) vs 78.4 (6.1) | 37 | NA | 44 (14) vs 41 (13) | MDT: cardiovascular research nurse, dietitian, geriatric cardiologist, physicians | Nurse-led, multidisciplinary intervention:   - - Intensive patient education about CHF and its treatment, individualized assessment, and instruction with reinforcement.   - Patient-centred consultation on discharge planning and care after discharge, medication analysis with specific recommendations and intensive follow-up after discharge, individualized home visits and telephone contact   Control group: conventional care with standard treatment | 3 | Hospital readmission, all-cause mortality |
| Riegel, 2006 (82) | USA | RCT | Community hospitals close to the US-Mexico border | 134 (Chronic HF, 78.4% <high school education)  IG: 69  CG: 65 | 72 (11) | 46.3 | III-IV | 43.2 (18.1) | Bilingual/bicultural Mexican American registered nurses, nurse case manager, physicians, research assistant | Telephone case management group:   - - Decision-support software program to guide nurse case manager.   - Standardise patient care management: patient self-care education, patients diet recommendations, medication adherence, telephone call reminder and documentation.   Control group: usual care | 6 | Hospital readmission, all-cause mortality |
| Rollman, 2021 (83) | USA | 3-arm, single blind, randomised clinical trial | 8 university-based and community hospitals in southwestern Pennsylvania | 756 (HF with LVEF <45%)  Enhanced UC: 252  Blended: 251  Usual care: 126  Without depression group: 127 | 64 (13) | 56 | II-IV | 28 (9.4) | Study cardiologist, nurse care managers, primary care physicians, psychiatrist | Enhanced usual care:   - - Nurse provided, telephone-delivered collaborative care program for HF.   - Counselling for HF self-care, scheduled follow-up, and usual care for depression.   - Regular case review meetings.   Blended:   - - Nurse provided, telephone-delivered collaborative care program for HF and depression.   - Counselling for HF self-care and depression.   - Regular case review meetings.   Usual care group:   - - Usual care for HF and depression.   Without depression group (non-randomized):   - - Usual care for HF   - Non-depressed comparison cohort | 12 | Rehospitalizations, mortality, change in medication |
| Sadik, 2005 (84) | UAE | RCT | General medical wards/cardiology/medical outpatient clinics in Al-Ain Hospital | 221 (HF)  IG: 109  CG: 112 | Mean= 58.6 | 50 | I-IV | NA | Research pharmacist, nurses, physicians | Intervention group:   - - Structured pharmaceutical care service, patient counselling and education (patient self-monitoring program and titration of medication) were given by research pharmacist.   - Pharmacist liaison with physicians   Control group: traditional services | 12 | Hospital admission, mortality |
| Sahlin, 2022 (85) | Sweden | Multicentre RCT | 5 primary care centres and 2 hospitals in southern Sweden | 118 (HF, 45% with EF<40%)  IG: 58  CG: 60 | IG vs CG:  80 (8) vs 77 (11) | 61 | I-IV | NA | Health care professional, research nurse | Intervention group:   - - Home-based self-care enhancement tool (OPTILOGG) which connected to a weight scale, incorporated symptom monitoring, interactive education, and adjustment of loop diuretics in the patient’s home.   Control group: standard care | 8­­­­ | ED visit, HF admission, mortality |
| Schwarz, 2008 (86) | USA | Pilot RCT | Tertiary teaching hospital in Northeastern Ohio | 102 (HF patient/caregiver dyads)  IG: 51  CG: 51 | IG vs CG: 77.1 (7.3) vs 79.1 (6.9) | 48 | II-IV | NA | RN data collector, nurse, APN, HF care manager, primary physician, cardiologist | Intervention group:   - - Usual care + daily telemonitoring with self-management of HF using electronic home monitoring (EHM) and data was transmitted electronically to HF care manager/APN for monitoring.   - Social support from home health care and EHM by an APN.   Control group: usual care | 3 | Hospital readmission, ED visit, mortality |
| Scuffham, 2017 (87) | Australia | Multicentre RCT | Tertiary hospitals | 787 (Chronic HF, 14% remote-dwelling)  IG: 396  CG: 391 | 74 (12) | 58.7 | III-IV | 31.4 (8.9) | HF-nurse, cardiology specialist | Intensified form of heart failure management programme (INT-HF-MP):   - - MDT management using Green Amber Red Delineation of rIsk And Need (GARDIAN).   - Combination of repeat home visits and STS (via an automated GARDIAN-ANGEL system to flag alerts and communications with the HF Nurse) were applied according to GARDIAN-HF Status:   - Red flag: weekly contacts for 12mo   - Yellow flag: weekly contacts until review at 6mo   - Green: routine 3 monthly calls   Control group: standard management | 12 | Change in medication, hospital readmission, all-cause mortality |
| Seto, 2012 (88) | Canada | RCT | University Health Network Heart Function Clinic | 100 (HF)  IG: 50  CG: 50 | IG vs CG: 55.1 (13.7) vs 52.3 (13.7) | 79 | II-IV | 27.1 (7.8) vs 27.0 (9.9) | Cardiologists, nurse practitioners | Telemonitoring group (6 months):   - - Standard care + daily telemonitoring of BP and body weight; weekly single-lead ECGs, and answered daily symptom questions on a mobile phone.   - Instructions were sent to the patients’ mobile phones and alerts to a cardiologist’s mobile phone as required.   Control group: standard care | 6 | Change in medication, mortality, hospital admission, ED visit |
| Sherwood, 2017 (89) | USA | Randomized comparative trial | Duke University Medical Centre, the UNC Health Care system, and the Durham VA Medical Centre | 180 (HF outpatients with reduced ejection fraction)  CST: 90  HFE: 90 | 57.7 (11.5) | 73 | II-III | 30.0 (9.6) | Physician’s assistant, clinical psychologist, interventionist | Telephone-based coping skills training (CST) intervention (16 weeks):   - - Delivered by a clinical psychologist, 16 weekly 30-minute individual phone calls to teach HF patients self-management skills and how to cope more effectively with psychological distress associated with heart failure.   Heart failure education (HFE):   - - 16 weekly telephone calls for extended (standardized) care on heart failure education. | 36 | All-cause mortality |
| Shively, 2005 (90) | USA | Single-site RCT | The Veterans Affairs (VA) San Diego Healthcare System | 116 (HF, 75% Caucasian)  IG: 58  CG: 58 | 67 (10) | 95 | I-IV | Median: 40% | Nurses | Behavioural management program (15 weeks)   - - Usual care + four classes and three phone calls   - Establishment of patients’ cognitive and behavioural skills, self-monitoring, goal setting and individualizing health life-style changes.   Control group: usual care | 16 | All-cause mortality |
| Sisk, 2006 (91) | USA | RCT | 4 hospitals in Harlem, New York | 406 (systolic dysfunction HF, 32.5% Hispanic adults, 36.7% aged ≥65 years)  IG: 203  CG: 203 | 59.4 (13.7) | 53.7 | I-IV | <40 | Bilingual nurse, clinician, internist, cardiologist | Nurse-led intervention (12 months):   - - Usual care + diet counselling, medication adherence and self-management of symptoms.   - Facilitation of evidence-based changes to medications in discussions with patients’ clinicians   - Initial visit and regular scheduled follow-up telephone calls.   Control group: usual care | 12 | Hospitalizations, ED visit, mortality, change in medication |
| Smith, 2014 (92) | USA | RCT | University of Kansas Medical Center | 198 (HF)  IG: 92  CG: 106 | 62.3 (13.2) | 62 | III-IV | 30 (16.1) | MDT: nurse practitioner, mental health clinical nurse specialist, social worker, dietician | Self-Management and Care of Heart Failure intervention group:   - - Standard care plus 4 weekly clinic appointments plus one booster clinic at month 6.   - MDT engaged patients in HF self-management skills.   Control group: standard care   - - Patient received HF education.   - Regular telephone/outpatient follow-up | 12 | Composite of hospitalization and death, change in medication |
| Srisuk, 2017 (93) | Thailand | RCT | 2 public hospitals in southern Thailand. | 100 (Patient-carer dyads)  IG: 50  CG: 50 | IG vs CG: 65 (14) vs 59 (18) | 47 | I-III | 51 (13) vs 50 (13) | Nurse, research personnel | Family-based education programme (6 months):   - - Face-to-face education counselling, HF manual and DVD and telephone support.   Control group: usual care   - - Standard medical and nursing care | 6 | Mortality |
| Stewart, 1999 (94) | Australia | RCT | Tertiary referral hospital | 200 (chronic HF)  IG: 100  CG: 100 | IG vs CG: 75.2 (7.1) vs 76.1 (9.3) | 62 | II-IV | 37 (10) vs 37 (11) | Cardiac nurse, primary care physician, cardiologist | Multidisciplinary home-based intervention:   - - Patients and their families received a combination of remedial counselling, introduction of strategies designed to improve treatment adherence and response, introduction of a simple exercise regimen, and incremental monitoring by family/carers.   - Home visits by a cardiac nurse after discharge, repeated if a patient had ≥ unplanned readmissions within 6 months of the index admission.   Control group: usual care | 6 | Hospital readmissions, mortality |
| Stewart, 2015 (95) | Australia | Pragmatic, single-centre, open-label RCT | Tertiary‐referral hospital with specialist cardiac services | 624 (chronic HF, cardiac inpatients, 70% CAD)  NIL-CHF: 310  CG: 314 | 66 (11) | 71 | NA | 61 (10) | Specialist cardiac nurse, community pharmacists, dietitians, diabetes educators, additional cardiac rehabilitation, or other allied healthcare services | Nurse-led Intervention for Less Chronic Heart Failure (NIL-CHF):   - - Short term (initial 6‐month period) comprehensive home visit at 7–14 days post‐index discharge   - Subsequent management was adjusted according to the GARDIAN tool.   - Longer term: subjects were able to contact the nurse for continued support and advice and visited the NIL‐CHF clinic at 18 and 36 months.   - Repeat home visits followed an unplanned hospitalization   Control group: standard care | 51.0 (8.2) | Composite of hospitalization and mortality, ED visit, change in medications |
| Thompson, 2005 (96) | UK | Cluster RCT | York District Hospital and Scunthorpe General Hospital in the North of England | 106 (CHF)  IG: 58  CG: 48 | IG vs CG: 73 (14) vs 72 (12) | 72.5 | III-IV | 31 (8) vs 29 (11) | GPs, study specialist nurses | Clinic plus home-based intervention (C+HBI) (6 months):   - - Patient education on diagnosis, symptom recognition, symptom management, lifestyle issues and crisis management.   - Patients were given specialist nurse contact.   Control group: standard care | 6 | Hospital readmission, all-cause mortality |
| Tsuchihashi-Makaya, 2013 (97) | Japan | RCT | 3 cardiology hospitals in Hokkaido | 168 (HF)  IG: 84  CG: 84 | IG vs CG: 76.9 (10.9) vs 75.8 (12.1) | 70 | I-III | 47.4 (16.6) vs 47.4 (15.7) | MDT: nurse, cardiologists, dietitian, and pharmacist | Home-based disease management intervention (6 months):   - - Usual care + home visits by nurses to provide symptom monitoring, education, counselling, and telephone follow-up by nurses and routine follow-up by cardiologists   Control group: usual care   - - Comprehensive discharge education using a booklet.   - Routine follow-up after discharge | 12 | All-cause mortality, HF-related hospitalization |
| Vaillant-Roussel, 2016 (98) | France | Cluster RCT | Hospitals in 4 areas of the Auvergne region | 241 (chronic HF)  IG: 115  CG: 126 | 74 (10.5) | 62 | I-III | 50.9 (13.2) vs 47.7 (15.2) | GPs | Intervention group (27 active GPs):   - - GPs were trained to manage their own education objectives (e.g., diet, treatment adherence) and patients’ objectives (e.g., to be able to walk their grandchildren to school)   - Regular patient education sessions every 3 months and discussion sessions with the GP   Control group (27 active GPs):   - - GPs attended a 3-hour information session to learn about the case report forms and the inclusion and exclusion criteria. | 19 | Hospitalization, mortality |
| Wagenaar, 2019 (99) | Netherlands | 3‐group parallel multicentre pragmatic RCT | 9 Dutch outpatient clinics | 450 (HF)  UC: 150  UC+HFM: 150  EACP: 150 | 66.8 (11.0) | 74.2 | I-IV | 35.7 (10.8) | Cardiologist, HF nurse specialists, healthcare workers at the HF outpatient clinic, GP | Usual care group (UC):   - - Average four routine consultations a year.   ‘Heartfailurematters.org’ website group (HFM):   - - Usual care + information on the use of the HFM website from the HF nurse.   - Participants received an informative leaflet + a reminder by e‐mail to use the website 3-monthly.   E‐health adjusted care pathway group (EACP):   - - UC + HFM intervention + the use of e‐Vita platform with telemonitoring facilities and patient self-management.   - HF nurses received an alert via the e‐Vita platform if abnormal data for further action; patients received monthly reminders by e‐mail. | 12 | Composite of hospitalization and mortality |
| Wakefield, 2008 (100) | USA | RCT | Veterans Affairs Medical Centre | 148 (CHF)  Telephone: 47  Videotape: 52  CG: 49 | 69.3 (9.6) | 99 | I-IV | 41.4 | Study nurse, physician | Telehealth-facilitated post-discharge support program:   - - Treatment plans: behavioural skill training strategies, self-monitoring strategies, self-efficacy enhancement strategies and external cognitive strategies.  1. Telephone: all intervention contacts were conducted using their personal telephone in their home. 2. Videophone: subjects used a system that consisted of a television monitor and a camera kit with a microphone and video camera.   Control group: usual care | 12 | Hospital admission, all-cause mortality |
| Weintraub, 2010 (101) | USA | Multicentre RCT | 3 in Massachusetts and 1 in Rhode Island | 188 (HF, 47% ischemic cardiomyopathy)  IG: 95  Comparator group: 93 | IG vs CG: 69.5 (14.2) vs 68.5 (12.8) | 66 | I-IV | 30 | Nurse manager, HF cardiologist, HF nurse, physician | Specialized Primary and Networked Care in Heart Failure (SPAN-CHF) disease management program plus the automated health monitoring (AHM) system (90 days):   - - Weekly phone calls to all actively enrolled patients to review clinical status   - Weekly conference with the HF team to review all actively enrolled patients   - 24/7 telephone access to a nurse manager   - Nurse managers accessed and reviewed AHM data daily basis and response if necessary.   Comparator group:   - - Subjects received SPAN-CHF disease management program. | 3 | Composite of hospitalizations, all-cause mortality |
| Wierzchowiecki, 2006 (102) | Poland | RCT | Karol Marcinkowski Medical University in Poznań, Franciszek Raszeja Community Hospital | 160 (chronic HF)  IG: 80  CG: 80 | IG vs CG: 67 (10.2) vs 69.5 (10.7) | 59.5 | II-IV | 36 (15) vs 35 (18) | MDT: a cardiologist, a heart failure nurse (HF nurse), a psychologist and a physiotherapist | Multidisciplinary care (MDC) (12 months):   - - Comprehensive education from the HF nurse and the cardiologist.   - Telephone counselling and home-based interventions by the HF nurse available daily.   - Regular follow-up visits / home visit for those patients with advanced HF and unable to travel to the clinic.   Control group: routine care | 12 | Composite of readmissions and mortality |
| Woodend, 2008 (103) | Canada | RCT | Hospital | 121 (HF)  IG: 62  CG: 59 | IG vs CG: 67 (13) vs 66 (11) | 72 | II-IV | NA | Community physician, cardiologist, nurse | Intervention arm (3 months):   - - Video conferences with a nurse, daily transmission of weight and BP; periodic transmission of 12-lead electrocardiogram   - Self-care education by the telehome-care nurse.   - Frequent conferences in the first few weeks after discharge and tapered over the 3-month period.   Control group: usual care | 12 | Hospital readmission |
| Wootton, 2009 (104) | Australia | RCT | Hospital in New South Wales | 409 (HF)  IG: 214  CG: 195 | IG vs CG: 82 (4) vs 83 (4) | 68.5 | NA | NA | GP (care coordinator), specialist nurses and call centre staff at McKesson Asia Pacific | Intervention group:   - - Telephone counselling, patient support (either by telephone or face-to-face) and provision of facts sheets.   - Education and promotion of lifestyle matters and education regarding medication taken.   Control group:   - - Standard information available from patients’ GPs and no counselling and support services via telephone were provided. | 12 | All-cause mortality |
| Young, 2016 (105) | USA | RCT | Rural critical access hospital in Nebraska | 105 (HF)  IG: 54  CG: 51 | 70.2 (12.2) | 36 | II-III | 55.7 (11.1) | Nurse | Intervention group (12 weeks):   - - Usual care + PATCH intervention 🡪 2 phases:   - A one-on-one-in-hospital SM training session   - Post-discharge reinforcement sessions delivered by telephone   - Self-management: workbooks, toolkits available   Control group: usual care   - - Standard discharge teaching for HF, regular follow-up. | 6 | Hospital readmission, ED visits |
| Yu, 2015 (106) | Hong Kong | Single-centre RCT | University-affiliated hospital in Hong Kong | 178 (Chronic HF)  IG: 90  CG: 88 | 78.6 (6.9) | 45 | II-IV | 41.1 (16.1) vs 39 (14.3) | Cardiac nurse | Transitional care group:   - - Home-based nursing intervention.   - Predischarge visit, two home visits, and regular telephone calls over 9 months to provide self-care education and support, optimized health surveillance, and facilitation in use of community services.   Control group: usual care | 9 | Mortality |

**Footnotes:** APN: advanced practice nurse; BP: blood pressure; BW: body weight; CAD: coronary artery disease; CBT: cognitive behaviour therapy; CDS: Cardiac depressions scores; CG: control group; CHF: congestive heart failure; COPD: chronic obstructive pulmonary disease; CR: cardiac rehabilitation; DM: diabetes mellitus; ED: emergency department; ENABLE CHF-PC: Educate, Nurture, Advise, Before Life Ends Comprehensive Heartcare for Patients and Caregivers; GARDIAN: Green Amber Red Delineation of rIsk And Need; GP: general practitioner; GUIDED-HF: Guidelines in Emergency Department Patients With Heart Failure; HF: heart failure; zHHW: Home-Heart-Walk; HNC: HeartNetCare; HR: heart rate; IG: intervention group; IQR: interquartile range; LVEF: left ventricular ejection fraction; INH: Interdisciplinary Network Heart Failure; MC: multidisciplinary care; MDT: multidisciplinary team; NA: not available; NYHA: New York Heart Association; PCT: Palliative Care Team; PNC: Palliative Care Nurse Coach; RA: research assistant; REACH-HF: Rehabilitation EnAblement in CHronicHeart Failure; RSP: Reciprocal Peer Support; sd: standard deviation; SMS: short message service; STS: structured telephone support; TM: telemonitoring; TNC: transition nurse coordinator; UAE: United Arab Emirates; UC: usual care; UK: United Kingdom; USA: United States of America

**Table S4.** Assessment of publication bias using the Egger's test

| **Outcomes** | **Number of trials** | **Eggar's test (p-value)** | **95% confidence interval** |
| --- | --- | --- | --- |
| All-cause hospital readmission | 60 | 0.066 | -1.06 to 0.04 |
| Heart failure-related hospital readmission | 40 | 0.327 | -0.97 to 0.33 |
| Cardiovascular-related readmission | 17 | 0.397 | -1.52 to 0.64 |
| All-cause mortality | 93 | 0.564 | -0.45 to 0.25 |
| Heart failure-related mortality | 5 | 0.444 | -1.36 to 0.77 |
| Cardiovascular-related mortality | 11 | 0.245 | -1.04 to 0.34 |

**Table S5.** Meta-analysis results of mortality and hospital readmission, stratified by individual quality improvement strategy

|  | All-cause hospital readmission | | | Heart failure-related hospital readmission | | | Cardiovascular-related hospital readmission | | |
| --- | --- | --- | --- | --- | --- | --- | --- | --- | --- |
| QI strategy | **N** | **RR (95% CI)** | **P-value** | **N** | **RR (95% CI)** | **P-value** | **N** | **RR (95% CI)** | **P-value** |
| Healthcare system | | | | | | | | | |
| Care management | 44 | 0.97 (0.92–1.02) | 0.180 | 31 | 0.84 (0.78–0.89) | <0.001 | 12 | 0.96 (0.85–1.09) | 0.510 |
| Team change | 33 | 0.91 (0.85–0.98) | 0.008 | 19 | 0.86 (0.80–0.93) | <0.001 | 7 | 0.90 (0.74–1.09) | 0.270 |
| Electronic patient registry | 5 | 1.01 (0.97–1.06) | 0.600 | 2 | 0.91 (0.82–1.02) | 0.090 | 2 | 1.08 (0.93–1.26) | 0.310 |
| Facilitated relay | 33 | 0.97 (0.92–1.02) | 0.270 | 23 | 0.84 (0.78–0.90) | <0.001 | 9 | 0.87 (0.79–0.96) | 0.007 |
| E-health | 26 | 0.98 (0.92–1.05) | 0.620 | 18 | 0.81 (0.75–0.88) | <0.001 | 8 | 0.88 (0.70–1.11) | 0.290 |
| Continuous QI | 33 | 0.97 (0.92–1.03) | 0.310 | 23 | 0.86 (0.79–0.94) | 0.001 | 12 | 0.94 (0.87–1.01) | 0.110 |
| Healthcare providers | | | | | | | | | |
| Audit and feedback | 5 | 0.92 (0.79–1.07) | 0.270 | 4 | 0.95 (0.81–1.11) | 0.540 | 1 | 1.07 (0.89–1.29) | 0.450 |
| Clinician education | 8 | 0.89 (0.79–1.00) | 0.040 | 4 | 0.94 (0.81–1.10) | 0.450 | 2 | 0.70 (0.31–1.60) | 0.400 |
| Clinician reminder | 11 | 0.98 (0.88–1.10) | 0.770 | 8 | 0.66 (0.47–0.93) | 0.020 | 5 | 0.75 (0.43–1.31) | 0.310 |
| Patients | | | | | | | | | |
| Patient education | 39 | 0.96 (0.91–1.02) | 0.210 | 22 | 0.84 (0.78–0.90) | <0.001 | 10 | 0.93 (0.85–1.02) | 0.110 |
| Promotion of self-management | 30 | 0.98 (0.92–1.04) | 0.490 | 21 | 0.83 (0.76–0.92) | <0.001 | 8 | 0.95 (0.86–1.05) | 0.300 |
| Patient reminder system | 9 | 0.92 (0.83–1.01) | 0.090 | 6 | 0.81 (0.65–1.00) | 0.050 | 2 | 1.06 (0.82–1.37) | 0.670 |
|  | **All-cause mortality** | | | **Heart failure-related mortality** | | | **Cardiovascular-related mortality** | | |
| QI strategy | **N** | **RR (95% CI)** | **P-value** | **N** | **RR (95% CI)** | **P-value** | **N** | **RR (95% CI)** | **P-value** |
| Healthcare system | | | | | | | | | |
| Care management | 69 | 0.90 (0.85–0.95) | <0.001 | 4 | 0.82 (0.52–1.27) | 0.370 | 8 | 0.74 (0.61–0.90) | 0.003 |
| Team change | 50 | 0.90 (0.85–0.96) | 0.001 | 2 | 0.62 (0.27–1.42) | 0.260 | 5 | 0.69 (0.51–0.94) | 0.020 |
| Electronic patient registry | 6 | 1.01 (0.88–1.15) | 0.900 | ─ | NA | ─ | 2 | 0.92 (0.66–1.28) | 0.630 |
| Facilitated relay | 54 | 0.87 (0.81–0.93) | <0.001 | 2 | 0.62 (0.27–1.42) | 0.260 | 7 | 0.73 (0.58–0.92) | 0.007 |
| E-health | 34 | 0.88 (0.81–0.96) | 0.003 | 2 | 0.91 (0.54–1.55) | 0.740 | 7 | 0.73 (0.58–0.91) | 0.005 |
| Continuous QI | 57 | 0.90 (0.84–0.96) | 0.001 | 2 | 1.00 (0.25–3.96) | 1.000 | 8 | 0.73 (0.59–0.90) | 0.003 |
| Healthcare providers | | | | | | | | | |
| Audit and feedback | 10 | 0.84 (0.61–1.15) | 0.270 | 1 | 0.99 (0.76–1.29) | 0.930 | 1 | 0.87 (0.59–1.30) | 0.510 |
| Clinician education | 11 | 0.93 (0.84–1.03) | 0.180 | 1 | 0.99 (0.76–1.29) | 0.930 | 0 | NA | ─ |
| Clinician reminder | 15 | 0.87 (0.75–1.00) | 0.040 | 1 | 1.00 (0.21–4.88) | 1.000 | 3 | 0.48 (0.24–0.95) | 0.040 |
| Patients | | | | | | | | | |
| Patient education | 52 | 0.90 (0.85–0.95) | <0.001 | 2 | 0.94 (0.73–1.22) | 0.650 | 8 | 0.74 (0.61–0.90) | 0.003 |
| Promotion of self-management | 46 | 0.90 (0.85–0.96) | <0.001 | 2 | 0.67 (0.31–1.43) | 0.300 | 5 | 0.75 (0.60–0.94) | 0.010 |
| Patient reminder system | 11 | 1.01 (0.88–1.15) | 0.770 | 2 | 0.91 (0.54–1.55) | 0.740 | 1 | 1.04 (0.57–1.90) | 0.900 |

Footnotes: E-health, electronic health; N, number; NA, not available; QI, quality improvement; RR, risk ratio. (Fixed-effects model was used when *I^2^*<50%, otherwise, random-effects model was used)

**References**

1. Lim LL, Lau ESH, Kong APS, Davies MJ, Levitt NS, Eliasson B, et al. Aspects of Multicomponent Integrated Care Promote Sustained Improvement in Surrogate Clinical Outcomes: A Systematic Review and Meta-analysis. Diabetes Care. 2018;41(6):1312-20.

2. Agvall B, Alehagen U, Dahlström U. The benefits of using a heart failure management programme in Swedish primary healthcare. Eur J Heart Fail. 2013;15(2):228-36.

3. Angermann CE, Störk S, Gelbrich G, Faller H, Jahns R, Frantz S, et al. Mode of action and effects of standardized collaborative disease management on mortality and morbidity in patients with systolic heart failure the interdisciplinary network for heart failure (INH) study. Circulation: Heart Failure. 2012;5(1):25-35.

4. Antonicelli R, Spazzafumo L, Scalvini S, Olivieri F, Matassini MV, Parati G, et al. Exercise: a "new drug" for elderly patients with chronic heart failure. Aging. 2016;8(5):860-72.

5. Arjunan P, Trichur RV. The Impact of Nurse-Led Cardiac Rehabilitation on Quality of Life and Biophysiological Parameters in Patients With Heart Failure: a Randomized Clinical Trial. Journal of nursing research. 2020.

6. Austin J, Williams R, Ross L, Moseley L, Hutchison S. Randomised controlled trial of cardiac rehabilitation in elderly patients with heart failure. European Journal of Heart Failure. 2005;7(3 SPEC. ISS.):411-7.

7. Bakitas MA, Dionne-Odom JN, Ejem DB, Wells R, Azuero A, Stockdill ML, et al. Effect of an Early Palliative Care Telehealth Intervention vs Usual Care on Patients With Heart Failure: The ENABLE CHF-PC Randomized Clinical Trial. JAMA Intern Med. 2020;180(9):1203-13.

8. Barker A, Barlis P, Berlowitz D, Page K, Jackson B, Lim WK. Pharmacist directed home medication reviews in patients with chronic heart failure: A randomised clinical trial. International Journal of Cardiology. 2012;159(2):139-43.

9. Bekelman DB, Plomondon ME, Carey EP, Sullivan MD, Nelson KM, Hattler B, et al. Primary Results of the Patient-Centered Disease Management (PCDM) for Heart Failure Study: a Randomized Clinical Trial. JAMA internal medicine. 2015;175(5):725‐32.

10. Bekelman DB, Allen LA, McBryde CF, Hattler B, Fairclough DL, Havranek EP, et al. Effect of a Collaborative Care Intervention vs Usual Care on Health Status of Patients With Chronic Heart Failure: The CASA Randomized Clinical Trial. JAMA Intern Med. 2018;178(4):511-9.

11. Berger R, Moertl D, Peter S, Ahmadi R, Huelsmann M, Yamuti S, et al. N-Terminal Pro-B-Type Natriuretic Peptide-Guided, Intensive Patient Management in Addition to Multidisciplinary Care in Chronic Heart Failure. A 3-Arm, Prospective, Randomized Pilot Study. Journal of the American College of Cardiology. 2010;55(7):645-53.

12. Bernocchi P, Vitacca M, La Rovere MT, Volterrani M, Galli T, Baratti D, et al. Home-based telerehabilitation in older patients with chronic obstructive pulmonary disease and heart failure: A randomised controlled trial. Age and Ageing. 2018;47(1):82-8.

13. Blum K, Gottlieb SS. The effect of a randomized trial of home telemonitoring on medical costs, 30-day readmissions, mortality, and health-related quality of life in a cohort of community-dwelling heart failure patients. Journal of Cardiac Failure. 2014;20(7):513-21.

14. Bocchi EA, Cruz F, Guimarães G, Pinho Moreira LF, Issa VS, Ayub Ferreira SM, et al. Long-term prospective, randomized, controlled study using repetitive education at six-month intervals and monitoring for adherence in heart failure outpatients: the REMADHE trial. Circulation Heart failure. 2008;1(2):115‐24.

15. Bouvy ML, Heerdink ER, Urquhart J, Grobbee DE, Hoes AW, Leufkens HG, et al. Effect of a pharmacist-led intervention on diuretic compliance in heart failure patients: a randomized controlled study. Journal of cardiac failure. 2003;9(5):404‐11.

16. Boyne JJ, Vrijhoef HJ, Crijns HJ, De Weerd G, Kragten J, Gorgels AP. Tailored telemonitoring in patients with heart failure: results of a multicentre randomized controlled trial. European journal of heart failure. 2012;14(7):791‐801.

17. Chen C, Li X, Sun L, Cao S, Kang Y, Hong L, et al. Post-discharge short message service improves short-term clinical outcome and self-care behaviour in chronic heart failure. ESC Heart Failure. 2019;6(1):164-73.

18. Chew HSJ, Sim KLD, Choi KC, Chair SY. Effectiveness of a nurse-led temporal self-regulation theory-based program on heart failure self-care: a randomized controlled trial. International journal of nursing studies. 2021;115:103872.

19. Cockayne S, Pattenden J, Worthy G, Richardson G, Lewin R. Nurse facilitated Self-management support for people with heart failure and their family carers (SEMAPHFOR): a randomised controlled trial. International journal of nursing studies. 2014;51(9):1207‐13.

20. Comín-Colet J, Enjuanes C, Verdú-Rotellar JM, Linas A, Ruiz-Rodriguez P, González-Robledo G, et al. Impact on clinical events and healthcare costs of adding telemedicine to multidisciplinary disease management programmes for heart failure: Results of a randomized controlled trial. Journal of telemedicine and telecare. 2016;22(5):282-95.

21. Dalal HM, Taylor RS, Jolly K, Davis RC, Doherty P, Miles J, et al. The effects and costs of home-based rehabilitation for heart failure with reduced ejection fraction: The REACH-HF multicentre randomized controlled trial. European Journal of Preventive Cardiology. 2019;26(3):262-72.

22. Dar O, Riley J, Chapman C, Dubrey SW, Morris S, Rosen SD, et al. A randomized trial of home telemonitoring in a typical elderly heart failure population in North West London: Results of the Home-HF study. European Journal of Heart Failure. 2009;11(3):319-25.

23. Davidson PM, Cockburn J, Newton PJ, Webster JK, Betihavas V, Howes L, et al. Can a heart failure-specific cardiac rehabilitation program decrease hospitalizations and improve outcomes in high-risk patients? European Journal of Cardiovascular Prevention and Rehabilitation. 2010;17(4):393-402.

24. de la Porte PWFB-A, Lok DJA, van Veldhuisen DJ, van Wijngaarden J, Cornel JH, Zuithoff NPA, et al. Added value of a physician-and-nurse-directed heart failure clinic: results from the Deventer-Alkmaar heart failure study. Heart (British Cardiac Society). 2007;93(7):819-25.

25. DeBusk RF, Miller NH, Parker KM, Bandura A, Kraemer HC, Cher DJ, et al. Care management for low-risk patients with heart failure: A randomized, controlled trial. Annals of Internal Medicine. 2004;141(8):606-13.

26. Del Sindaco D, Pulignano G, Minardi G, Apostoli A, Guerrieri L, Rotoloni M, et al. Two-year outcome of a prospective, controlled study of a disease management programme for elderly patients with heart failure. Journal of cardiovascular medicine (Hagerstown, Md). 2007;8(5):324‐9.

27. Dendale P, De Keulenaer G, Troisfontaines P, Weytjens C, Mullens W, Elegeert I, et al. Effect of a telemonitoring-facilitated collaboration between general practitioner and heart failure clinic on mortality and rehospitalization rates in severe heart failure: the TEMA-HF 1 (TElemonitoring in the MAnagement of Heart Failure) study. Eur J Heart Fail. 2012;14(3):333-40.

28. Dessie G, Burrowes S, Mulugeta H, Haile D, Negess A, Jara D, et al. Effect of a self-care educational intervention to improve self-care adherence among patients with chronic heart failure: a clustered randomized controlled trial in Northwest Ethiopia. BMC cardiovascular disorders. 2021;21(1):374.

29. DeWalt DA, Malone RM, Bryant ME, Kosnar MC, Corr KE, Rothman RL, et al. A heart failure self-management program for patients of all literacy levels: A randomized, controlled trial [ISRCTN11535170]. BMC Health Services Research. 2006;6.

30. Dewalt DA, Schillinger D, Ruo B, Bibbins-Domingo K, Baker DW, Holmes GM, et al. Multisite randomized trial of a single-session versus multisession literacy-sensitive self-care intervention for patients with heart failure. Circulation. 2012;125(23):2854-62.

31. Doughty RN, Wright SP, Pearl A, Walsh HJ, Muncaster S, Whalley GA, et al. Randomized, controlled trial of integrated heart failure management: The Auckland heart failure management study. European Heart Journal. 2002;23(2):139-46.

32. Du H, Newton PJ, Budhathoki C, Everett B, Salamonson Y, Macdonald PS, et al. The Home-Heart-Walk study, a self-administered walk test on perceived physical functioning, and self-care behaviour in people with stable chronic heart failure: A randomized controlled trial. Eur J Cardiovasc Nurs. 2018;17(3):235-45.

33. Dunbar SB, Reilly CM, Gary R, Higgins MK, Culler S, Butts B, et al. Randomized clinical trial of an integrated self-care intervention for persons with heart failure and diabetes: quality of life and physical functioning outcomes. Journal of cardiac failure. 2015;21(9):719‐29.

34. Ekman I, Andersson B, Ehnfors M, Matejka G, Persson B, Fagerberg B. Feasibility of a nurse-monitored, outpatient-care programme for elderly patients with moderate-to-severe, chronic heart failure. European Heart Journal. 1998;19(8):1254-60.

35. Ekman I, Fagerberg B, Andersson B, Matejka G, Persson B. Can treatment with angiotensin-converting enzyme inhibitors in elderly patients with moderate to severe chronic heart failure be improved by a nurse-monitored structured care program? A randomized controlled trial. Heart Lung. 2003;32(1):3-9.

36. Elpida K, Constantinos PH, Ioannis V, Athena K, Sotirios K, Eleftherios K, et al. A constructivist approach to teaching patients with heart failure: results from an intervention study. European journal of cardiovascular nursing. 2020.

37. Freedland KE, Carney RM, Rich MW, Steinmeyer BC, Rubin EH. Cognitive behavior therapy for depression and self-care in heart failure patients a randomized clinical trial. JAMA Internal Medicine. 2015;175(11):1773-82.

38. Gattis WA, Hasselblad V, Whellan DJ, O'Connor CM. Reduction in heart failure events by the addition of a clinical pharmacist to the heart failure management team: results of the Pharmacist in Heart Failure Assessment Recommendation and Monitoring (PHARM) Study. Arch Intern Med. 1999;159(16):1939-45.

39. Investigators G. Randomised trial of telephone intervention in chronic heart failure: DIAL trial. bmj. 2005;331(7514):425.

40. Güder G, Störk S, Gelbrich G, Brenner S, Deubner N, Morbach C, et al. Nurse-coordinated collaborative disease management improves the quality of guideline-recommended heart failure therapy, patient-reported outcomes, and left ventricular remodelling. European Journal of Heart Failure. 2015;17(4):442-52.

41. Harrison MB, Browne GB, Roberts J, Tugwell P, Gafni A, Graham ID. Quality of life of individuals with heart failure: a randomized trial of the effectiveness of two models of hospital-to-home transition. Medical care. 2002;40(4):271‐82.

42. Heisler M, Halasyamani L, Cowen ME, Davis MD, Resnicow K, Strawderman RL, et al. Randomized controlled effectiveness trial of reciprocal peer support in heart failure. Circulation Heart failure. 2013;6(2):246‐53.

43. Jaarsma T, Halfens R, Tan F, Abu-Saad HH, Dracup K, Diederiks J. Self-care and quality of life in patients with advanced heart failure: the effect of a supportive educational intervention. Heart & lung. 2000;29(5):319‐30.

44. Jiménez-Marrero S, Yun S, Cainzos-Achirica M, Enjuanes C, Garay A, Farre N, et al. Impact of telemedicine on the clinical outcomes and healthcare costs of patients with chronic heart failure and mid-range or preserved ejection fraction managed in a multidisciplinary chronic heart failure programme: A sub-analysis of the iCOR randomized trial. Journal of telemedicine and telecare. 2020;26(1-2):64-72.

45. Jolly K, Taylor RS, Lip GYH, Davies M, Davis R, Mant J, et al. A randomized trial of the addition of home-based exercise to specialist heart failure nurse care: The Birmingham Rehabilitation Uptake Maximisation study for patients with Congestive Heart Failure (BRUM-CHF) study. European Journal of Heart Failure. 2009;11(2):205-13.

46. Kalter-Leibovici O, Freimark D, Freedman LS, Kaufman G, Ziv A, Murad H, et al. Disease management in the treatment of patients with chronic heart failure who have universal access to health care: a randomized controlled trial. BMC Med. 2017;15(1):90.

47. Kasper EK, Gerstenblith G, Hefter G, Van Anden E, Brinker JA, Thiemann DR, et al. A randomized trial of the efficacy of multidisciplinary care in heart failure outpatients at high risk of hospital readmission. Journal of the American College of Cardiology. 2002;39(3):471‐80.

48. Kenealy TW, Parsons MJ, Rouse AP, Doughty RN, Sheridan NF, Hindmarsh JK, et al. Telecare for diabetes, CHF or COPD: effect on quality of life, hospital use and costs. A randomised controlled trial and qualitative evaluation. PLoS One. 2015;10(3):e0116188.

49. Köberich S, Lohrmann C, Mittag O, Dassen T. Effects of a hospital-based education programme on self-care behaviour, care dependency and quality of life in patients with heart failure--a randomised controlled trial. Journal of clinical nursing. 2015;24(11-12):1643-55.

50. Koehler F, Winkler S, Schieber M, Sechtem U, Stangl K, Böhm M, et al. Impact of remote telemedical management on mortality and hospitalizations in ambulatory patients with chronic heart failure: the telemedical interventional monitoring in heart failure study. Circulation. 2011;123(17):1873-80.

51. Koehler F, Koehler K, Deckwart O, Prescher S, Wegscheider K, Kirwan BA, et al. Efficacy of telemedical interventional management in patients with heart failure (TIM-HF2): a randomised, controlled, parallel-group, unmasked trial. The Lancet. 2018;392(10152):1047-57.

52. Koehler J, Stengel A, Hofmann T, Wegscheider K, Koehler K, Sehner S, et al. Telemonitoring in patients with chronic heart failure and moderate depressed symptoms: results of the Telemedical Interventional Monitoring in Heart Failure (TIM-HF) study. Eur J Heart Fail. 2021;23(1):186-94.

53. Kotooka N, Kitakaze M, Nagashima K, Asaka M, Kinugasa Y, Nochioka K, et al. The first multicenter, randomized, controlled trial of home telemonitoring for Japanese patients with heart failure: home telemonitoring study for patients with heart failure (HOMES-HF). Heart and Vessels. 2018;33(8):866-76.

54. Laramee AS, Levinsky SK, Sargent J, Ross R, Callas P. Case management in a heterogeneous congestive heart failure population: A randomized controlled trial. Archives of Internal Medicine. 2003;163(7):809-17.

55. Liljeroos M, Ågren S, Jaarsma T, Årestedt K, Strömberg A. Long term follow-up after a Randomized integrated educational and psychosocial intervention in patient-partner dyads affected by heart failure. PLoS ONE. 2015;10(9).

56. Lowrie R, Mair FS, Greenlaw N, Forsyth P, Jhund PS, McConnachie A, et al. Pharmacist intervention in primary care to improve outcomes in patients with left ventricular systolic dysfunction. European Heart Journal. 2012;33(3):314-24.

57. López Cabezas C, Falces Salvador C, Cubí Quadrada D, Arnau Bartés A, Ylla Boré M, Muro Perea N, et al. Randomized clinical trial of a postdischarge pharmaceutical care program vs. regular follow-up in patients with heart failure. Farmacia Hospitalaria. 2006;30(6):328-42.

58. Mehralian H, Salehi S, Moghaddasi J, Amiri M, Rafiei H. The comparison of the effects of education provided by nurses on the quality of life in patients with congestive heart failure (CHF) in usual and home-visit cares in Iran. Global journal of health science. 2014;6(3):256‐60.

59. Mejhert M, Kahan T, Persson H, Edner M. Limited long term effects of a management programme for heart failure. Heart (british cardiac society). 2004;90(9):1010‐5.

60. Murray MD, Young J, Hoke S, Tu W, Weiner M, Morrow D, et al. Pharmacist intervention to improve medication adherence in heart failure: A randomized trial. Annals of Internal Medicine. 2007;146(10):714-25.

61. Nahlén Bose C, Persson H, Björling G, Ljunggren G, Elfström ML, Saboonchi F. Evaluation of a Coping Effectiveness Training intervention in patients with chronic heart failure - a randomized controlled trial. Eur J Cardiovasc Nurs. 2016;15(7):537-48.

62. Naylor MD, Brooten DA, Campbell RL, Maislin G, McCauley KM, Schwartz JS. Transitional Care of Older Adults Hospitalized with Heart Failure: A Randomized, Controlled Trial. Journal of the American Geriatrics Society. 2004;52(5):675-84.

63. Nolan RP, Ross HJ, Farkouh ME, Huszti E, Chan S, Toma M, et al. Automated E-Counseling for Chronic Heart Failure: cHF-CePPORT Trial. Circulation Heart failure. 2021;14(1):e007073.

64. Norman JF, Kupzyk KA, Artinian NT, Keteyian SJ, Alonso WS, Bills SE, et al. The influence of the HEART Camp intervention on physical function, health-related quality of life, depression, anxiety and fatigue in patients with heart failure. European Journal of Cardiovascular Nursing. 2020;19(1):64-73.

65. Nucifora G, Albanese MC, De Biaggio P, Caliandro D, Gregori D, Goss P, et al. Lack of improvement of clinical outcomes by a low-cost, hospital-based heart failure management programme. Journal of Cardiovascular Medicine. 2006;7(8):614-22.

66. O'Connor CM, Whellan DJ, Lee KL, Keteyian SJ, Cooper LS, Ellis SJ, et al. Efficacy and safety of exercise training in patients with chronic heart failure: HF-ACTION randomized controlled trial. Jama. 2009;301(14):1439-50.

67. Oddone EZ, Weinberger M, Giobbie-Hurder A, Landsman P, Henderson W. Enhanced access to primary care for patients with congestive heart failure. Veterans Affairs Cooperative Study Group on Primary Care and Hospital Readmission. Effective clinical practice : ECP. 1999;2(5):201-9.

68. Olivari Z, Giacomelli S, Gubian L, Mancin S, Visentin E, Di Francesco V, et al. The effectiveness of remote monitoring of elderly patients after hospitalisation for heart failure: The renewing health European project. International Journal of Cardiology. 2018;257:137-42.

69. Ong MK, Romano PS, Edgington S, Aronow HU, Auerbach AD, Black JT, et al. Effectiveness of Remote Patient Monitoring After Discharge of Hospitalized Patients With Heart Failure: The Better Effectiveness After Transition -- Heart Failure (BEAT-HF) Randomized Clinical Trial. JAMA Intern Med. 2016;176(3):310-8.

70. Ortiz-Bautista C, Morán-Fernández L, Díaz-García M, Delgado-Nicolás M, Ponz-de Antonio I, Rodríguez-Chaverri A, et al. Evaluation of a nurse-led intervention program in heart failure: A randomized trial. Med Clin (Barc). 2019;152(11):431-7.

71. Østergaard B, Mahrer-Imhof R, Wagner L, Barington T, Videbæk L, Lauridsen J. Effect of family nursing therapeutic conversations on health-related quality of life, self-care and depression among outpatients with heart failure: A randomized multi-centre trial. Patient Education and Counseling. 2018;101(8):1385-93.

72. Otsu H, Moriyama M. Effectiveness of an educational self-management program for outpatients with chronic heart failure. Japan journal of nursing science : JJNS. 2011;8(2):140-52.

73. Panella M, Marchisio S, Demarchi ML, Manzoli L, Di Stanislao F. Reduced in-hospital mortality for heart failure with clinical pathways: the results of a cluster randomised controlled trial. Qual Saf Health Care. 2009;18(5):369-73.

74. Pekmezaris R, Nouryan CN, Schwartz R, Castillo S, Makaryus AN, Ahern D, et al. A Randomized Controlled Trial Comparing Telehealth Self-Management to Standard Outpatient Management in Underserved Black and Hispanic Patients Living with Heart Failure. Telemedicine journal and e-health. 2019;25(10):917‐25.

75. Peters-Klimm F, Campbell S, Müller-Tasch T, Schellberg D, Gelbrich G, Herzog W, et al. Primary care-based multifaceted, interdisciplinary medical educational intervention for patients with systolic heart failure: lessons learned from a cluster randomised controlled trial. Trials. 2009;10:68.

76. Peters-Klimm F, Campbell S, Hermann K, Kunz CU, Müller-Tasch T, Szecsenyi J. Case management for patients with chronic systolic heart failure in primary care: the HICMan exploratory randomised controlled trial. Trials. 2010;11:56.

77. Philbin EF, Rocco TA, Lindenmuth NW, Ulrich K, McCall M, Jenkins PL. The results of a randomized trial of a quality improvement intervention in the care of patients with heart failure. The MISCHF Study Investigators. American journal of medicine. 2000;109(6):443‐9.

78. Piette JD, Striplin D, Marinec N, Chen J, Trivedi RB, Aron DC, et al. A Mobile Health Intervention Supporting Heart Failure Patients and Their Informal Caregivers: A Randomized Comparative Effectiveness Trial. J Med Internet Res. 2015;17(6):e142.

79. Powell LH, Calvin Jr JE, Richardson D, Janssen I, Mendes De Leon CF, Flynn KJ, et al. Self-management counseling in patients with heart failure: The heart failure adherence and retention randomized behavioral trial. JAMA - Journal of the American Medical Association. 2010;304(12):1331-8.

80. Rahimi K, Nazarzadeh M, Pinho-Gomes AC, Woodward M, Salimi-Khorshidi G, Ohkuma T, et al. Home monitoring with technology-supported management in chronic heart failure: A randomised trial. Heart. 2020;106(20):1573-8.

81. Rich MW, Beckham V, Wittenberg C, Leven CL, Freedland KE, Carney RM. A multidisciplinary intervention to prevent the readmission of elderly patients with congestive heart failure. New England Journal of Medicine. 1995;333(18):1190-5.

82. Riegel B, Carlson B, Glaser D, Romero T. Randomized Controlled Trial of Telephone Case Management in Hispanics of Mexican Origin With Heart Failure. Journal of Cardiac Failure. 2006;12(3):211-9.

83. Rollman BL, Anderson AM, Rothenberger SD, Abebe KZ, Ramani R, Muldoon MF, et al. Efficacy of Blended Collaborative Care for Patients With Heart Failure and Comorbid Depression: a Randomized Clinical Trial. JAMA internal medicine. 2021;181(10):1369‐80.

84. Sadik A, Yousif M, McElnay JC. Pharmaceutical care of patients with heart failure. Br J Clin Pharmacol. 2005;60(2):183-93.

85. Sahlin D, Rezanezad B, Edvinsson ML, Bachus E, Melander O, Gerward S. Self-care Management Intervention in Heart Failure (SMART-HF): A Multicenter Randomized Controlled Trial. Journal of cardiac failure. 2022;28(1):3‐12.

86. Schwarz KA, Mion LC, Hudock D, Litman G. Telemonitoring of heart failure patients and their caregivers: a pilot randomized controlled trial. Progress in cardiovascular nursing. 2008;23(1):18-26.

87. Scuffham PA, Ball J, Horowitz JD, Won C, Newton PJ, Macdonald P, et al. Standard vs. intensified management of heart failure to reduce healthcare costs: Results of a multicentre, randomized controlled trial. European Heart Journal. 2017;38(30):2340-8.

88. Seto E, Leonard KJ, Cafazzo JA, Barnsley J, Masino C, Ross HJ. Mobile phone-based telemonitoring for heart failure management: a randomized controlled trial. Journal of medical Internet research. 2012;14(1):e31.

89. Sherwood A, Blumenthal JA, Koch GG, Hoffman BM, Watkins LL, Smith PJ, et al. Effects of Coping Skills Training on Quality of Life, Disease Biomarkers, and Clinical Outcomes in Patients with Heart Failure: A Randomized Clinical Trial. Circulation: Heart Failure. 2017;10(1).

90. Shively M, Kodiath M, Smith TL, Kelly A, Bone P, Fetterly L, et al. Effect of behavioral management on quality of life in mild heart failure: A randomized controlled trial. Patient Education and Counseling. 2005;58(1):27-34.

91. Sisk JE, Hebert PL, Horowitz CR, McLaughlin MA, Wang JJ, Chassin MR. Effects of nurse management on the quality of heart failure care in minority communities: A randomized trial. Annals of Internal Medicine. 2006;145(4):273-83.

92. Smith CE, Piamjariyakul U, Wick JA, Spertus JA, Russell C, Dalton KM, et al. Multidisciplinary group clinic appointments: the Self-Management and Care of Heart Failure (SMAC-HF) trial. Circulation Heart failure. 2014;7(6):888‐94.

93. Srisuk N, Cameron J, Ski CF, Thompson DR. Randomized controlled trial of family-based education for patients with heart failure and their carers. Journal of advanced nursing. 2017;73(4):857‐70.

94. Stewart S, Marley JE, Horowitz JD. Effects of a multidisciplinary, home-based intervention on unplanned readmissions and survival among patients with chronic congestive heart failure: a randomised controlled study. Lancet. 1999;354(9184):1077-83.

95. Stewart S, Chan YK, Wong C, Jennings G, Scuffham P, Esterman A, et al. Impact of a nurse-led home and clinic-based secondary prevention programme to prevent progressive cardiac dysfunction in high-risk individuals: The Nurse-led Intervention for Less Chronic Heart Failure (NIL-CHF) randomized controlled study. European Journal of Heart Failure. 2015;17(6):620-30.

96. Thompson DR, Roebuck A, Stewart S. Effects of a nurse-led, clinic and home-based intervention on recurrent hospital use in chronic heart failure. European journal of heart failure. 2005;7(3):377‐84.

97. Tsuchihashi-Makaya M, Matsuo H, Kakinoki S, Takechi S, Kinugawa S, Tsutsui H. Home-based disease management program to improve psychological status in patients with heart failure in Japan. Circulation Journal. 2013;77(4):926-33.

98. Vaillant-Roussel H, Laporte C, Pereira B, De Rosa M, Eschalier B, Vorilhon C, et al. Impact of patient education on chronic heart failure in primary care (ETIC): a cluster randomised trial. BMC family practice. 2016;17:80.

99. Wagenaar KP, Broekhuizen BDL, Jaarsma T, Kok I, Mosterd A, Willems FF, et al. Effectiveness of the European Society of Cardiology/Heart Failure Association website ‘heartfailurematters.org’ and an e-health adjusted care pathway in patients with stable heart failure: results of the ‘e-Vita HF’ randomized controlled trial. European Journal of Heart Failure. 2019;21(2):238-46.

100. Wakefield BJ, Ward MM, Holman JE, Ray A, Scherubel M, Burns TL, et al. Evaluation of home telehealth following hospitalization for heart failure: A randomized trial. Telemedicine and e-Health. 2008;14(8):753-61.

101. Weintraub A, Gregory D, Patel AR, Levine D, Venesy D, Perry K, et al. A multicenter randomized controlled evaluation of automated home monitoring and telephonic disease management in patients recently hospitalized for congestive heart failure: the SPAN-CHF II trial. J Card Fail. 2010;16(4):285-92.

102. Wierzchowiecki M, Poprawski K, Nowicka A, Kandziora M, Piatkowska A, Jankowiak M, et al. A new programme of multidisciplinary care for patients with heart failure in Poznań: one-year follow-up. Kardiol Pol. 2006;64(10):1063-70; discussion 71-2.

103. Woodend AK, Sherrard H, Fraser M, Stuewe L, Cheung T, Struthers C. Telehome monitoring in patients with cardiac disease who are at high risk of readmission. Heart and Lung: Journal of Acute and Critical Care. 2008;37(1):36-45.

104. Wootton R, Gramotnev H, Hailey D. A randomized controlled trial of telephone-supported care coordination in patients with congestive heart failure. Journal of Telemedicine and Telecare. 2009;15(4):182-6.

105. Young L, Hertzog M, Barnason S. Effects of a home-based activation intervention on self-management adherence and readmission in rural heart failure patients: The PATCH randomized controlled trial. BMC Cardiovascular Disorders. 2016;16(1).

106. Yu DS, Lee DT, Stewart S, Thompson DR, Choi KC, Yu CM. Effect of Nurse-Implemented Transitional Care for Chinese Individuals with Chronic Heart Failure in Hong Kong: a Randomized Controlled Trial. Journal of the american geriatrics society. 2015;63(8):1583‐93.
